# Thirty biologically interpretable clusters of transcription factors distinguish cancer type

**DOI:** 10.1186/s12864-018-5093-z

**Published:** 2018-10-11

**Authors:** Zachary B. Abrams, Mark Zucker, Min Wang, Amir Asiaee Taheri, Lynne V. Abruzzo, Kevin R. Coombes

**Affiliations:** 1 Department of Biomedical Informatics, The Ohio State University, 1800 Cannon Drive, Columbus, 43210 OH USA; 2 Mathematical Biosciences Institute, The Ohio State University, 1735 Neil Avenue, Columbus, 43210 OH USA; 3 Department of Pathology, The Ohio State University, 129 Hamilton Hall, 1645 Neil Avenue, Columbus, 43210 OH USA

**Keywords:** TCGA, Pan-cancer, Clustering, Thresher, Gene expression

## Abstract

**Background:**

Transcription factors are essential regulators of gene expression and play critical roles in development, differentiation, and in many cancers. To carry out their regulatory programs, they must cooperate in networks and bind simultaneously to sites in promoter or enhancer regions of genes. We hypothesize that the mRNA co-expression patterns of transcription factors can be used both to learn how they cooperate in networks and to distinguish between cancer types.

**Results:**

We recently developed a new algorithm, Thresher, that combines principal component analysis, outlier filtering, and von Mises-Fisher mixture models to cluster genes (in this case, transcription factors) based on expression, determining the optimal number of clusters in the process. We applied Thresher to the RNA-Seq expression data of 486 transcription factors from more than 10,000 samples of 33 kinds of cancer studied in The Cancer Genome Atlas (TCGA). We found that 30 clusters of transcription factors from a 29-dimensional principal component space were able to distinguish between most cancer types, and could separate tumor samples from normal controls. Moreover, each cluster of transcription factors could be either (i) linked to a tissue-specific expression pattern or (ii) associated with a fundamental biological process such as cell cycle, angiogenesis, apoptosis, or cytoskeleton. Clusters of the second type were more likely also to be associated with embryonically lethal mouse phenotypes.

**Conclusions:**

Using our approach, we have shown that the mRNA expression patterns of transcription factors contain most of the information needed to distinguish different cancer types. The Thresher method is capable of discovering biologically interpretable clusters of genes. It can potentially be applied to other gene sets, such as signaling pathways, to decompose them into simpler, yet biologically meaningful, components.

**Electronic supplementary material:**

The online version of this article (10.1186/s12864-018-5093-z) contains supplementary material, which is available to authorized users.

## Background

Transcription factors (TF) are proteins that bind to DNA and control the rate of transcription for a set of genes; they are some of the most important regulators of gene expression [1]. In particular, they play a crucial role in development, differentiation, and the maintenance of cell type [2]. Furthermore, about one-third of TFs are tissue-specific [3], and TFs are over-represented among oncogenes [4]. Because of the vital role of TFs in the regulation of multiple critical biological processes, we hypothesize that the expression patterns of transcription factors contain sufficient information to distinguish between different types of cancer.

In order for TFs to carry out their regulatory programs, they must cooperate by forming networks [1]. Gaining a better understanding of how TFs cooperate to regulate gene expression can help us gain deeper insight into human genetics and disease, especially cancer. In order to identify cooperating TF networks, some researchers have clustered TFs according to known function or disease association [5, 6]. Others have focused on clustering TF binding sites by looking for common sequence motifs [7, 8]. Still other studies have applied clustering algorithms to patterns of TF protein expression [9, 10]. These studies are motivated by the observation that, essentially by definition, TFs working in concert must bind (to the same or to nearby binding sites, possibly exhibiting similar motifs) *at the same time* [11]. In other words, cooperating sets of TFs tend to be expressed together so that TF coexpression may be an effective proxy for cooperativity [12, 13]. To understand which TFs cooperate (and thus distinguish tissue types and cancer types), we propose to cluster them into biologically meaningful sets based on their coexpression at the mRNA level.

Clustering “features” (genes, proteins, transcription factors, etc.) is a core research problem in biomedical informatics [14–16]. The ability to group biological features into distinct *biologically interpretable* clusters would solve many important but challenging research problems, such as the identification of multi-dimensional biomarkers. The challenges posed by these research problems result in part from the nature of omics research, which has dramatically increased the feature space in many biomedical domains [17]. For this reason, grouping and clustering problems are more prevalent than ever and require more creative and robust solutions. In addition, as researchers increasingly look for more complex patterns in omics data, ensuring the biological interpretability of results is an increasingly important task [18].

**Fig. 1:**
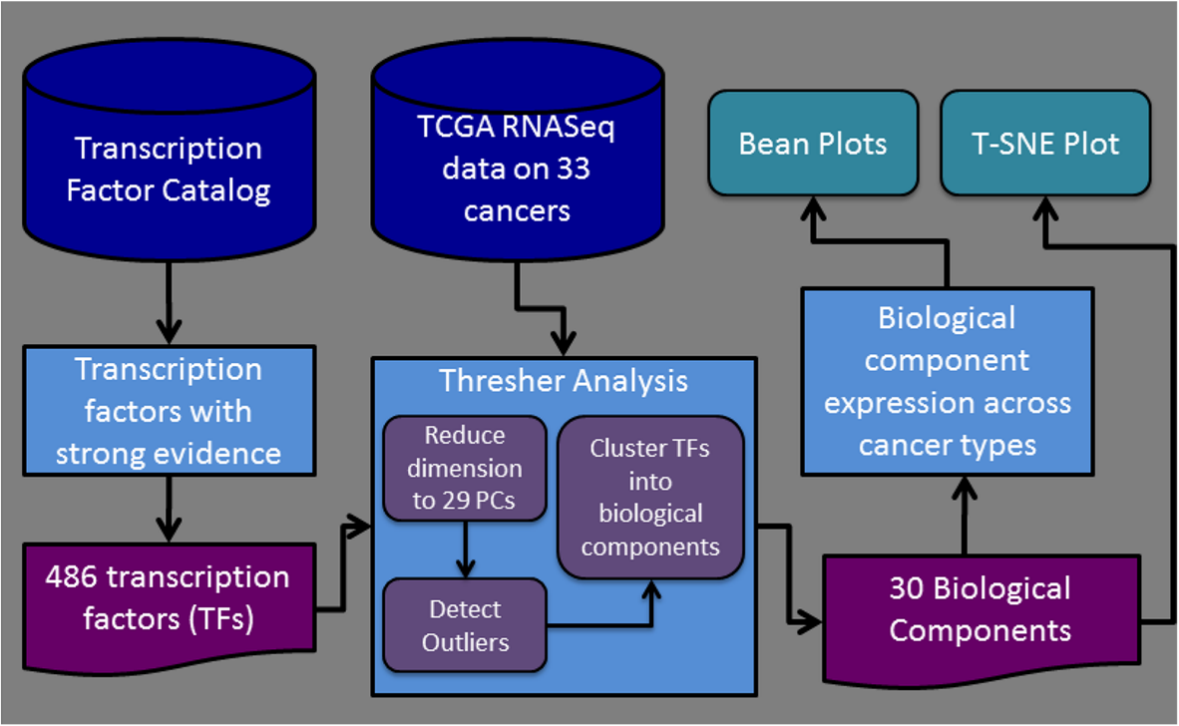
Workflow diagram showing the main analysis steps and results of the paper (TF = transcription factor; PC = principal component)

In this article, we apply a novel solution to the problem of clustering transcription factors; Fig. 1 illustrates the worflow. We demonstrate the ability of our recently described algorithm, Thresher [19], to cluster transcription factors into biologically interpretable one-dimensional clusters. Thresher employs concepts from principal component analysis, outlier filtering, and von Mises-Fisher mixture models. It is specifically designed both to determine the optimal number of clusters after filtering out insignificant “outlier” features and to replace the purely mathematical principal components with biologically relevant and interpretable clusters. We apply Thresher to the set of more than 10,000 RNA-Seq gene expression profiles of 33 kinds of cancers taken from The Cancer Genome Atlas (TCGA) [20]. We show that the expression patterns of 486 transcription factors in this dataset can be summarized by 29 principal components that are capable of distinguishing almost all of the cancer types assayed by TCGA, including separating cancer samples from the adjacent normal tissue. We further show that the 29 mathematical principal components can be replaced naturally by 30 clusters, which we call “*biological components*.” Each biological component has its own internal and coherent biological meaning. About 40% of the biological components appear to be directly related to a specific tissue type, while the other 60% are related to fundamental biological processes such as the cell cycle, angiogenesis, or apoptosis. We believe that Thresher’s ability to replace principal components with biologically interpretable components will have broad applicability.

## Results

### Number of principal components

We performed principal components analysis (PCA) on the dataset containing expression measurements of 486 transcription factors, as listed in the Transcription Factor Catalog [21], in 10,446 samples from studies of 33 different kinds of cancer in The Cancer Genome Atlas. The numbers of samples per cancer type are listed in Table 1. In order to estimate the number of significant components, we used the PCDimension R package [22], which implements automatic rules for the graphical Bayesian method introduced by Auer and Gervini [23]. The Auer-Gervini model uses a family of exponentially decaying prior distributions parametrized by a variable called *Θ* that controls the decay rate; they showed that the maximum a posteriori (MAP) estimate of the number of components is a non-increasing step function of *Θ*. In Additional file 1: Figure S1, we have plotted this step function for the TCGA transcription factor data.

**Table 1:** Number of samples per cancer type

Cancer code	Cancer type	N
ACC	Adrenocortical carcinoma	79
BLCA	Bladder Urothelial Carcinoma	427
BRCA	Breast invasive carcinoma	1212
CESC	Cervical squamous cell carcinoma and endocervical adenocarcinoma	309
CHOL	Cholangiocarcinoma	45
COAD	Colon adenocarcinoma	328
DLBC	Diffuse Large B-cell Lymphoma	48
ESCA	Esophageal carcinoma	196
GBM	Glioblastoma multiforme	171
HNSC	Head and Neck squamous cell carcinoma	566
KICH	Kidney Chromophobe	91
KIRC	Kidney renal clear cell carcinoma	606
KIRP	Kidney renal papillary cell carcinom	323
LAML	Acute Myeloid Leukemia	173
LGG	Brain Lower Grade Glioma	530
LIHC	Liver hepatocellular carcinoma	423
LUAD	Lung adenocarcinoma	576
LUSC	Lung squamous cell carcinoma	552
MESO	Mesothelioma	87
OV	Ovarian serous cystadenocarcinoma	307
PAAD	Pancreatic adenocarcinom	183
PCPG	Pheochromocytoma and Paraganglioma	187
PRAD	Prostate adenocarcinoma	550
READ	Rectum adenocarcinoma	105
SARC	Sarcoma	265
SKCM	Skin Cutaneous Melanoma	473
STAD	Stomach adenocarcinoma	450
TGCT	Testicular Germ Cell Tumor	156
THCA	Thyroid carcinoma	568
THYM	Thymoma	122
UCEC	Uterine Corpus Endometrial Carcinoma	201
UCS	Uterine Carcinosarcoma	57
UVM	Uveal Melanoma	80

In their paper, Auer and Gervini advise looking at this plot and selecting the “highest step that is long” to define the number of components. In our paper, we examined a variety of rules for automating this selection, including

- “Twice Mean”, in which any step that is longer than twice the mean step length is viewed as long;
- “CPT”, in which we first sort the steps by the length in increasing order, and then apply the “At Most One Change” algorithm implemented by the cpt.mean function in the changepoint R package to detect the first change point; and
- “Kmeans3”, in which we apply the K-means algorithm with *K*=3 to cluster the step lengths into small, medium, and large, where both “medium” and “large” are viewed as long.

In the simulation studies [22], we found that the first two of these methods, in particular, were competitive with the best existing techniques to estimate the number of components. When applying these methods to the transcription factor data, CPT claims that there are four components; Kmeans3 claims that there are 18, and Twice Mean claims that there are 29.

### Principal components distinguish cancer types

**Fig. 2:**
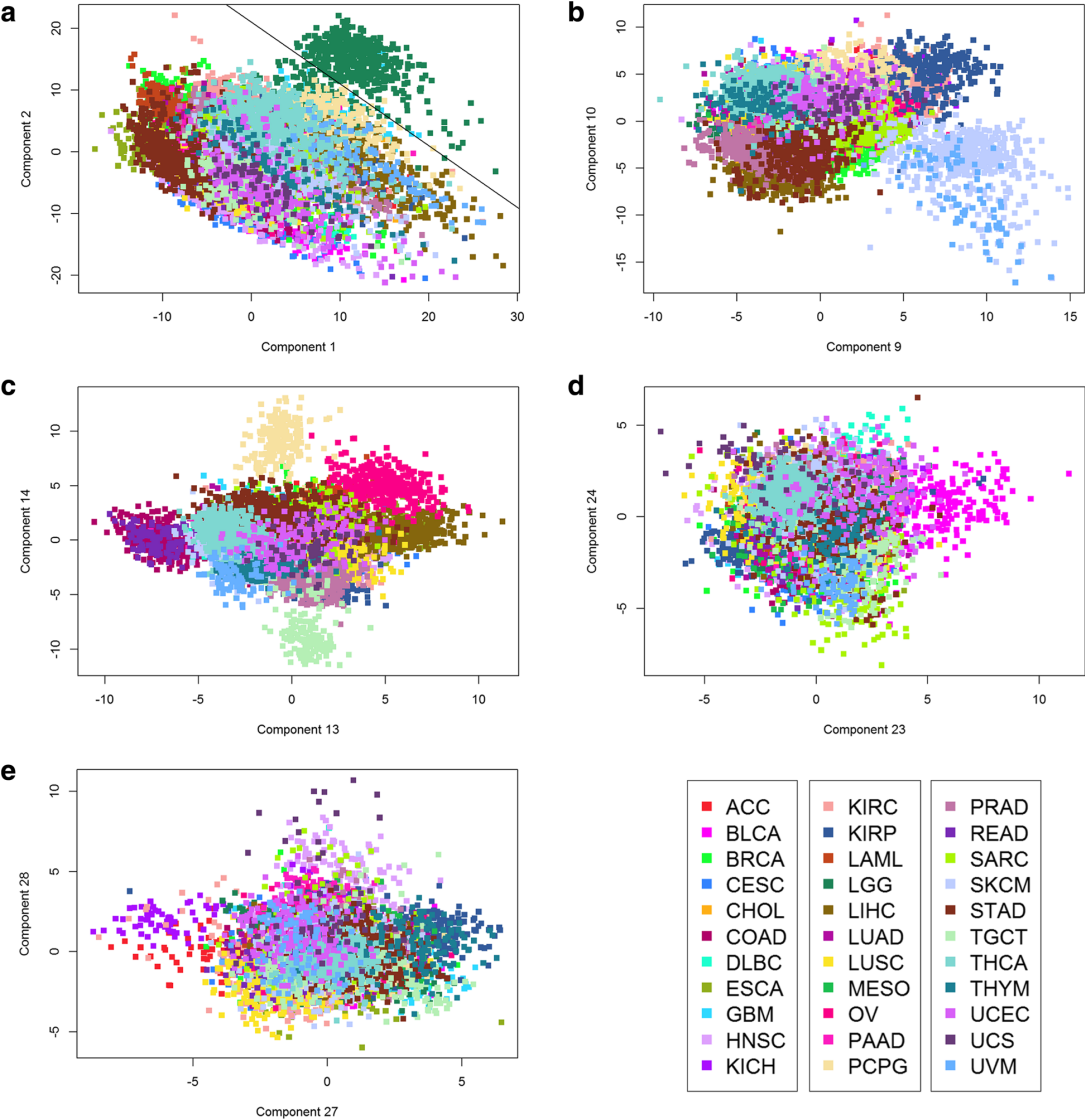
Principal component plots based on the expression patterns of 486 transcription factors. (Panel **a**: Principal components 1 and 2; **b**: Components 9 and 10; **c**: Components 13 and 14; **d**: Components 23 and 24; **e**: Components 27 and 28

To test visually whether the Twice Mean estimate of 29 significant principal components is reasonable, we prepared pairwise plots of different components. Some of these plots are shown in Fig. 2; a more extensive set is contained in Additional file 1: Figures S2–S15. In each plot, samples are colored by cancer type according to the color scheme shown in the bottom right panel. In panel (a) of Fig. 2, we show PCs 1 and 2. The “jade” samples in the upper right are low-grade gliomas (LGG). In panel (b), PCs 9 and 10, the two different shades of blue at the lower right come from samples of uveal or cutaneous skin melanomas (UVM; SKCM). In panel (c), PCs 13 and 14, the “pale yellow” samples at the top are pheochromocytoma and paraganglioma (PCPG) cancers. The “pale green” at the bottom are testicular germ cell tumors (TGCT). In panel (d), PCs 23 and 24, the “magenta” samples at the right are bladder cancer (BLCA) and the “yellowish green” at the bottom are sarcomas (SARC). In panel (e), PCs 27 and 28, the purple samples at the left are kidney chromophobe (KICH), one of three types of kidney cancer studied in TCGA, and the “red” samples are adrenocortical carcinomas (ACC). The “turquoise” samples at the right are thymomas (THYM). These figures support the conclusion that the principal components, at last including components 23–28 as claimed by the “Twice Mean” algorithm, contain information that helps distinguish different cancer types.

### Information to distinguish most cancer types is present in 29 principal components

Linear projections, such as those implemented in PCA, do not always give an accurate picture of how well-separated subgroups really are in high-dimensional spaces. In order to obtain more accurate visualizations, we applied the method of t-distributed stochastic neighbor embedding (t-SNE) [24, 25]. The results are shown in Fig. 3. In this figure, primary tumors are plotted with an open circle, metastases with a hollow triangle, and normal samples with an asterisk. This plot reveals the following results:

1. In almost every case, samples from one kind of cancer are well separated from other kinds.

**Fig. 3:**
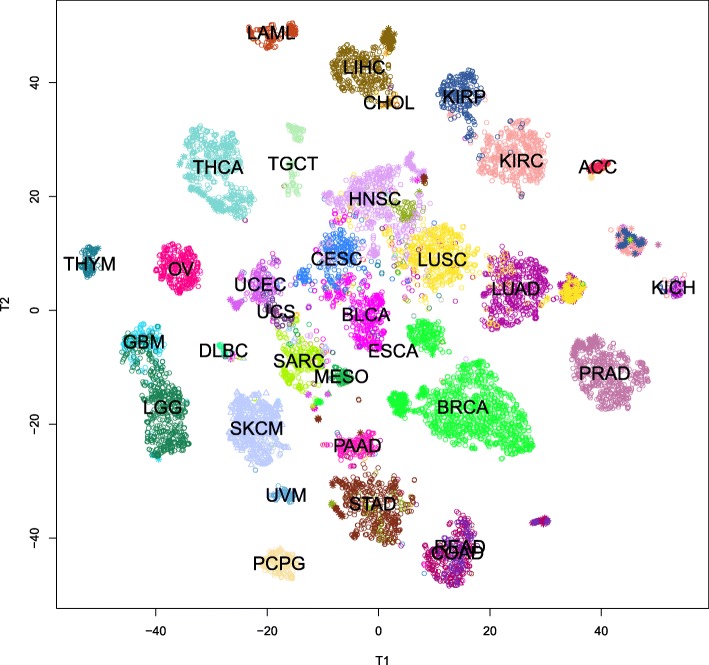
Plot of the non-linear t-SNE map of samples from 29-dimensional principal component space into two dimensions. Colors are the same as in the legend shown in Fig. 2. Primary tumors are plotted with an open circle, metastases with a hollow triangle, and normal samples with an asterisk
2. However, colon cancer (COAD) and rectal cancer (READ) are essentially indistinguishable. (See the bottom of the figure, right of center).
3. Moreover, normal samples of COAD or READ can be distinguished from tumors, but not from each other.
4. The two types of lung cancer (LUAD and LUSC, right of center) can mostly be distinguished, although there are a few samples that unexpectedly overlap the other group.
5. However, all the normal lung samples cluster together.
6. Even though one type of primary kidney cancer (KICH, far right) is very unlike the other two types (KIRC and KIRP, to the upper right), their normal samples cluster together.
7. There are clearly two very different subtypes of esophogeal cancer (ESCA). One clusters with the stomach cancers (STAD; bottom center) while the other clusters with the head-and-neck cancers (HNSC; center).
8. Most of the time, we can tell normal samples from primary tumors. In addition to the lung, kidney, and colorectal cancers that we have already mentioned, we can also separate subclusters of normal samples for thyroid (THCA; upper left), liver (LIHC, top center), prostate (PRAD, right), and breast (BRCA, lower right).
9. Breast cancer is also interesting, in that there are clearly at least two well-separated subtypes of breast cancer. The smaller set consists of triple negative breast cancer cases.

### Finding biological components

In addition to the fact that linear projections in PCA may not reveal the full extent of the separation of subtypes in high-dimensional spaces, the components themselves are difficult to interpret biologically. Whenever we use genes to cluster samples, the individual PCs are comprised of weighted linear combinations of genes. These combinations are chosen to maximize the percentage of variance explained and to satisfy the mathematically desirable property of orthogonality. In situations where many different biological processes may be at work, however, each PC often turns out to combine the effects of multiple processes.

To address this problem, we applied a new method, Thresher, that we recently developed [19]. The Thresher algorithm has three steps:

1. Use the PCDimension package [22] to determine the number *D* of significant principal components. Then we can view each gene (or transcription factor) as a vector of weights in the principal component space of dimension *D*.
2. The magnitude, or length, of these vectors is used to identify and remove outliers. Our simulations suggest that vectors of length <0.3 are safe to remove [19].
3. The remaining genes are then clustered based on the directions of their weight vectors. Equivalently, this process converts each gene into a point on a hypersphere in PC space. To cluster such points, we model the data using a mixture of von Mises-Fisher distributions [26, 27]. We assume that the number *K* of clusters satisfies *D*≤*K*≤2*D* and use the Akaike Information Criterion (AIC) to select the optimal *K*.

We want to emphasize two key points about the last step in this process. First, we are replacing the mathematical principal components, which are chosen to satisfy orthogonality, with more natural directions defined by the actual genes. For this reason, we refer to these clustered direction-vectors as “biological components.” Second, we allow the number *K* of biological components to be up to twice as large as the number *D* of principal components. The motivation driving this decision is that we want to separate genes whose expression patterns are negatively correlated. Such genes point in opposite directions in principal component space, and so they do not increase the mathematical dimension of the space.

When we applied Thresher to the TCGA transcription factor data, no outliers were found, and the mixture model concluded that there were a total of 30 clusters of transcription factors. Additional file 2: Table S0 lists the transcription factors belonging to each cluster. We then considered the data from each cluster separately. In each case, we found that the cluster spanned a one-dimensional principal component space (Additional file 1: Figures S16–S45). Moreover, the weights of the cluster members in the first principal component all had the same sign and were of roughly comparable magnitudes. Thus, we concluded that we had identified 30 sets (clusters) of transcription factors that tended to work together across more than 10,000 samples.

### Computation time

Operations were timed on an Intel ^*Ⓡ*^ i7-3930 CPU at 3.2 GHz running Windows ^*Ⓡ*^ 7 SP1. Performing PCA and using PCDimension to compute the number of components took 15 s. Running t-SNE took 93 s. Running Thresher took 256 s; however, this measurement includes automatically running the algorithm twice, once before and once after removing outliers. Each run also includes running the PCDimension code.

### Characterizing biological components

We hypothesized that each transcription factor cluster (or biological component) implements a single biological process. We used three different bioinformatics approaches to test this hypothesis and thus to annotate the biological entity associated with each biological component.

1. We prepared “bean plots” [28] of the average expression of each biological component in the TCGA samples, separated and colored by cancer type (Figs. 4, 5 and Additional file 1: Figures S46–S75).

**Fig. 4:**
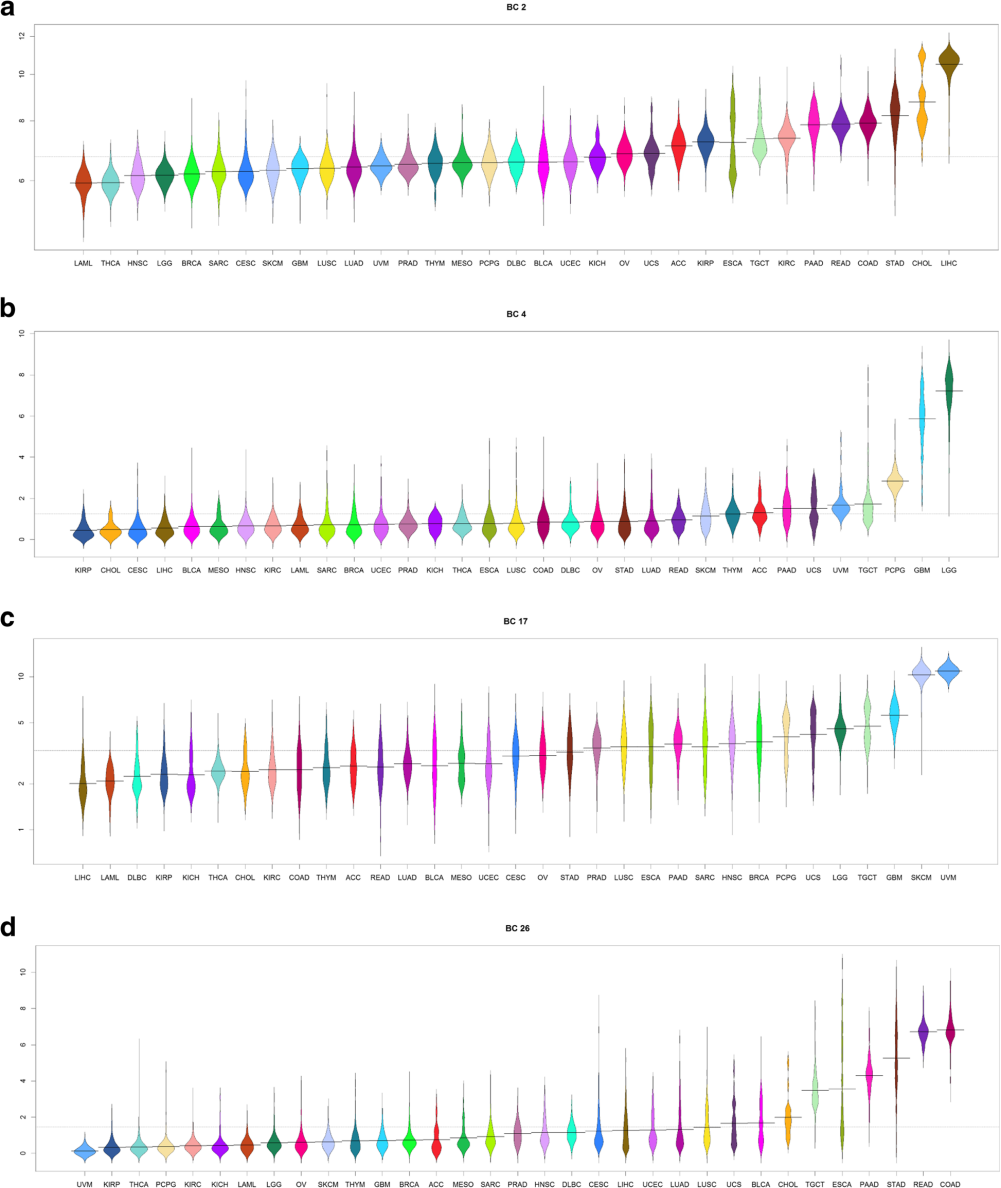
Bean plots of the expression of several “biological components” associated with tissue type. **a** Liver. **b** Brain. **c** Melanocytes. **d** Intestine

**Fig. 5:**
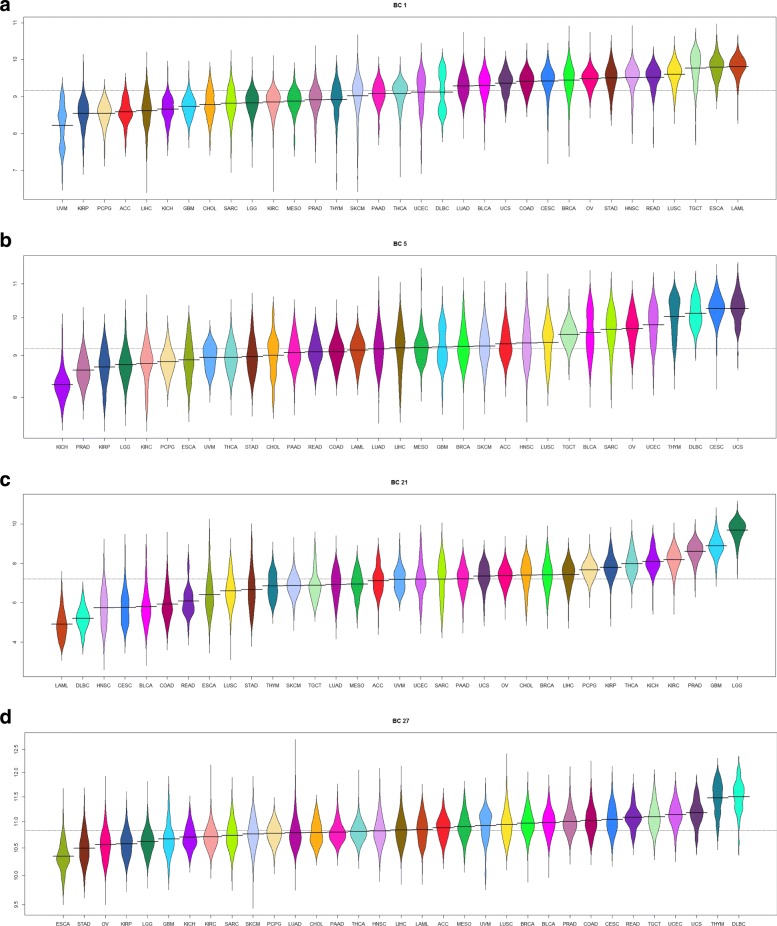
Bean plots of the expression of several “biological components” associated with embryonically lethal mouse phenotypes. **a** Cell cycle. **b** Cell cycle. **c** Cytoskeleton. **d** Ribosomes and endoplasmic reticulum
2. We identified the UniGene cluster corresponding to each transcription factor [29, 30]. We found the tissues listed as “cDNA sources” for the UniGene cluster, and for each biological component, recorded the tissues that appeared the maximal number of times.
3. We computed Pearson correlation coefficients between each of the 30 biological components and all 20,289 genes measured by RNA sequencing in the TCGA samples. For each biological component, we took the list of genes whose absolute correlation was at least 0.5 and uploaded it to the ToppGene website in order to perform gene set analyses [31].

A summary of the results of these analyses is shown in Table 2. More complete results are contained in Additional file 3: Tables S1–S30. We found that 12/30 (40%) of the biological components appeared to be associated with a specific tissue type. Four examples of the 12 tissue-specific components are shown in Fig. 4. The remaining 18/30 (60%) of the components were associated with fundamental biological processes, including cell cycle, angiogenesis, apoptosis, mitochondria, ribosomes, and the endoplastic reticulum. Eight of these eighteen biological components were also associated with “embryonically lethal” mouse phenotypes; four examples of the eight “embryonically lethal” biological-process components are illustrated in Fig. 5.

**Table 2:** Interpretations of biological components

Cl.	ToppGene	Lethal	Top five	UniGene tissues
1	Cell cycle; chromosome organization	Yes	LAML; ESCA; TGCT; LUSC; READ	More than four
2	Lipid metabolism; liver; cytochrome p450	No	LIHC; CHOL; STAD; COAD; READ	Brain; intestine; liver
3	Extracellular matrix; angiogenesis	No	UCEC; OV; UCS; SARC; PCPG	More than four
4	Synaptic signaling; neuron; synapse;	No	LGG; GBM; PCPG; TGCT; UVM	Brain
5	Cell cycle; condensed chromosome	Yes	UCS; CESC; DLBC; THYM; UCEC	More than four
6	Transcription factor activity; mitochondria	No	UVM; LAML; ESCA; LGG; STAD	More than four
7	Cell cycle	Yes	TGCT; UCS; OV; ESCA; READ	More than four
8	Mitochondrial; post-synapse; guanyl-nucleotide activity; lung; breast; ovary; testis	No	LGG; KIRC; SARC; LAML; GBM	Brain; lung; testis
9	Neuropeptide; synaptic signaling; abnormal pterygopalatine ganglion morphology	No	PCPG; PAAD; TGCT; READ; UCS	Brain
10	Transcription factor activity; centrosome; microtubule	No	LGG; GBM; UCS; TGCT; THYM	Brain; embryonic tissue; testis
11	Apoptosis; gametogenesis	No	LAML; OV; TGCT; UCS; UCEC	Brain; kidney; testis
12	Microtubule; centriole; stem cell	No	UVM; THYM; UCS; LGG; UCEC	Lung; prostate; uterus
13	Cytokine receptor activity; immune response; lymphadenopathy	No	DLBC; STAD; LAML; PAAD; ESCA	Intestine; lung; uterus
14	Regionalization; vertebral transformation; leukemia	No	KICH; KIRP; UCS; KIRC; PCPG	Kidney; uterus
15	Collagen; thyroid; thyroid hormone synthesis	No	THCA; LUAD; LUSC; KIRP; OV	Brain; lung; prostate; uterus
16	Muscle	No	UCS; HNSC; TGCT; OV; SARC	Muscle
17	Melanin; melanosome	No	UVM; SKCM; GBM; TGCT; LGG	Embryonic tissue
18	Epithelium development; abnormal digestive system development; keratinization	No	PRAD; BRCA; LUAD; LUSC; BLCA	Lung
19	Extracellular matrix; epithelium development; collagens; keratins	No	HNSC; UCS; CESC; ESCA; LUSC	Eye; lung; skin; uterus
20	Eye lens; reproduction; gametogenesis; pluripotent stem cells; TGCT	Yes	TGCT; ACC; THYM; OV; LAML	Embryonic tissue; testis
21	Cytoskeleton; tubulin binding; cell cycle; neuron;	Yes	LGG; GBM; PRAD; KIRC; KICH	Brain; eye; testis
22	Pol II; uveitis; abnormla myeloid cell morphology; ovary; trachea; lung	No	HNSC; ESCA; PAAD; STAD; CESC	More than four
23	NADH dehydrogenase activity; oxidative phosphorylation; respiratory electron transport;	Yes	ACC; THYM; PCPG; UVM; THCA	More than four
24	NADH dehydrogenase activity; oxidative phosphorylation; developing kidney	Yes	LAML; ESCA; STAD; BRCA; OV	More than four
25	Immune response; leukocyte activation;	No	DLBC; LAML; STAD; THYM; LUAD	Blood; brain; lung; lymph node
26	Fatty acid binding; dogestion; microvillus; intestinal epithelium	No	COAD; READ; STAD; PAAD; ESCA	Intestine
27	Structural constituent of ribosome; endoplasmic reticulum; eukaryotic translation	Yes	DLBC; THYM; UCS; UCEC; TGCT	More than four
28	CNS development; growth cone; forebrain;	No	GBM; LGG; UCS; CESC; HNSC	Brain
29	Growth factor binding; angiogenesis; focal adhesion;	Yes	ESCA; HNSC; GBM; LUSC; KIRC	Brain; embryonic tissue; lung
30	Cell-cell adhesion; cell-cell junction; digestive system; claudins	No	STAD; READ; COAD; PAAD; BLCA	Lung; pancreas

## Discussion

### Expression of transcription factors separates cancer types

We began by testing the hypothesis that transcription factor expression could differentiate cancers in the TCGA dataset. The results displayed in the nonlinear t-SNE map (Fig. 3) clearly demonstrate that using 30 biological components derived from 486 transcription factors produced a clear separation between most TCGA cancer types. This map illustrates the relative separation or biological distance between cancer types based on transcription factor expression. This visualization displays a more explicit separation between cancer types than any of the principal component plots alone, thus producing important biological insights not observable through simpler linear methods.

Our initial observation is that cancer types that occur in the same or similar tissues or organ systems may be difficult to distinguish. Both low-grade gliomas (LGG) and glioblastomas (GBM), for example, occur in the brain. These two diseases are plotted near each other in Fig. 3; in fact, they overlap slightly. Moreover, the transcription factor clustering groups them closer to each other than to any other cancer. This grouping is understandable given that some of the biological components are specific for transcription factors expressed in the brain. Other examples include rectal adenocarcinoma entirely overlapping with colon adenocarcinomas, both uterine cancers clustering together, and some esophageal cancers overlapping stomach cancers.

The examples from the previous paragraph might lead one to suspect that the separation we are seeing is driven not by cancer type but by baseline differences in TF expression in the tissues where the cancers originate. However, there is evidence from other cancer types that tissue type alone does not completely explain the results. For example, TCGA studied three different types of kidney cancer, and there are four associated clusters in the t-SNE map. Two of these clusters appear next to each other at the right center of the map; they represent kidney renal clear cell carcinoma (KIRC) and kidney renal papillary cell carcinoma (KIRP). The other two clusters also appear next to each other, but in the middle of the bottom portion of the map. One of these contains samples of normal kidney coming from all three studies. The final cluster contains all of the kidney chromophobe (KICH) cases, along with a few KIRC and KIRP cases. The relative positions of the three types of kidney cancer are consistent with recent reports that KIRC and KIRP samples are similar to proximal tubule segments, whereas KICH samples are more similar to distal segments [32–34].

Samples derived from lung tissue display a similar phenomenon. TCGA studied both lung adenocarcinoma (LUAD) and lung squamous cell carcinoma (LUSC) histological subtypes. Our results find three clusters of lung-derived samples that represent (in order, lying on a ray emanating from the center of the figure) LUSC, LUAD, and normal lung. In particular, (1) normal samples cluster together, (2) normal samples are separate from either cancer group, and (3) the squamous cell and adenocarcinomas are clearly distinct. It also suggests that transcription factor expression in normal lung tissue may be more similar to lung adenocarcinoma than to lung squamous cell carcinoma. These findings are consistent with the fact that the two histologies of lung cancer arise from different cell types. LUSC arises from the squamous epithelium that lines the airways and alveoli, while LUAD arises from the more numerous glandular or alveolar type 2 cells [35–37].

The distinction between squamous cell carcinomas and adenocarcinomas is present throughout Fig. 3. Adenocarcinomas (including prostate (PRAD), colon (COAD), lung (LUAD), pancreas (PAAD), ovarian (OV), stomach (STAD), and some esophagus (ESCA) tumors) appear to be scattered around the periphery of the map. By contrast, squamous cell carcinomas (including lung (LUSC), cervix (CESC), head and neck (HNSC), and esophagus (ESCA)) cluster near each other, regardless of the organ system, in the center of the map. This observation suggests an underlying similarity in the transcription factor expression profiles of the squamous cell cancers regardless of the tissue type of squamous cell cancer.

Breast cancer (BRCA) illustrates a different phenomenon. Most samples are in one large cluster, with normal samples in a distinct small separate cluster nearby. However, the triple negative cases form a completely independent cluster separate from either the normal samples or the main cluster of breast cancer samples. This indicates that triple negative breast cancer, in terms of transcription factor expression, represents a distinct and completely separate form of breast cancer. Using the transcription factor components that separate these triple negative cases may prove useful in treating triple negative breast cancer patients through a better understanding of the underlying molecular biology.

In every cancer study where TCGA has included normal controls, the t-SNE map shows that the normal samples differ from the tumors. In most cases, they form a completely separate cluster. In others, like prostate (PRAD), thyroid (THCA), or bladder (BLCA), they can be found on the periphery of the tumor cluster. This differentiation shows that transcription factor expression alone is able to differentiate cancer from the adjacent normal tissue. This is of particular importance due to its potential applications in translational medicine and potential use in cancer screenings.

Other research groups have already applied t-SNE and related methodologies to TCGA data in order to separate different types of cancer [38–41]. Those studies used the entire transcriptome of 20,000 genes, unlike our study that restricts itself to only 486 transcription factors. In every case, our findings using only TFs are similar to the results from these previous studies. Significantly, the inability to (fully) separate certain pairs of cancers, such as COAD/READ and UCS/UCEC, was seen previously by researchers using the whole transcriptome [39]. This finding shows that our inability to separate those cancers does not occur because we only used TFs. Overall, the consistency between our results and previous whole transcriptome pan-cancer studies strengthens the underlying hypothesis that transcription factors may be the primary driver for the differentiation between different cancer types in various tissue types.

### Biological components

We used Thresher to cluster transcription factors according to a transformation of their expression into 30 one-dimensional biological components. We then hypothesized that each biological component was associated with a particular biological process. Examining the biology underlying each of the 30 components revealed two general categories of transcription factor clusters: 12 were tissue specific and 18 were biological function specific. Among the 18 function-associated clusters, 8 were also associated with embryonically lethal mouse phenotypes. The tissue specific components consist of transcription factors produced only within the cancers arising from that tissue type. In embryonic lethal components, the transcription factors were part of universally expressed pathways such as the cell cycle. Examples of tissue specific pathways are shown in Fig. 4. It is clear that certain cancers have a significantly higher expression of a particular cluster of transcription factors relative to other cancers. This makes biological sense, as biological processes peculiar to a given tissue type would be expected to be specifically altered in cancer specific to that tissue.

Figure 5 further validates this pattern in the context of constitutive or embryonic lethal components. In these cases there is little, or no difference of expression between cancer types since the transcription factors that make up these components are *comparably* expressed across all tissue types, a requirement for self-viability. Thus it is the tissue specific components, and especially those that differentiate between normal and cancerous samples within a specific cancer and those that differentiate between two cancers in the same organ system, that are of particular clinical utility and interest as biomarkers.

Overall, these patterns demonstrate Thresher’s effectiveness at clustering genes by expression. The fact that transcription factor clusters associated with biological processes necessary for viability show similar expression levels across cancers is an important validation. Additionally, our finding that differentiation between transcription factor clusters tends to correspond to differentiation of cases (whether they are cancer or normal samples), or by the type or tissue of origin, as well as by biological process, indicates that our method yields clustering patterns that correspond to real underlying biological differences.

## Conclusion

Transcription factors play a vital role in regulating gene expression. By applying the Thresher method, we were able to summarize the activity of 486 transcription factors using only 30 distinct biological components. Analyzing these components helps us better understand how transcription factors interact with each other in regulatory networks. Moreover, the expression data summarized by this small set of biological components was sufficient to distinguish most of the different cancer types and to separate tumors from normal controls within cancer types. This suggests that patterns in these biological components may be useful in understanding the underlying biology of cancers. Additionally, since transcription factors are common targets for treatment, these patterns may also be useful for identifying viable genes to target in new treatments or in developing treatment regimens for various subtypes of cancer.

The methodology that combines Thresher with t-SNE maps should be broadly applicable. It can, in principle, be used to understand the regulatory control that microRNAs and methylation have on gene and protein expression. It can also be applied to other biologically meaningful subsets of genes than transcription factors; obvious candidates for future study include sets of genes that are known to interact in signaling pathways or in the regulation of mechanisms like apoptosis.

## Methods

### Data sources

The data used in our experiments comes from The Cancer Genome Atlas (TCGA). The TCGA RNASeq data was selected because it (1) is publicly available, (2) contains a large number of samples, and (3) contains many different cancers and thus tissue types. Data were downloaded from the FireBrowse portal [42], one cancer type at a time, on 2016-09-21. The number of samples per cancer type are listed in Table 1.

The list of human transcription factors was downloaded from the Transcription Factor Catalog [21] on 2017-10-18 after conducting a search for “TF Gene”. We only retained 486 genes that were annotated in the database to have “strong” evidence of transcription factor activity. Since TCGA contained all 486 transcription factors, the final data set contained 486 rows (transcription factors) and 10,446 coiumns (patient samples).

### Statistical methods

All analyses were performed in version 3.4.3 of the R Statistical Programming Environment [43]. Computations and timings were performed on a computer with an Intel ^*Ⓡ*^ Core™ i7-3930K CPU at 3.20 Ghz and 32 GB of RAM, running Microsoft ^*Ⓡ*^ Windows ^*Ⓡ*^ 7 Professional SP1.

The t-distributed stochastic neighbor embedding (t-SNE) algorithm uses a non-linear dimension reduction method that enables visualization on a two-dimensional scatter plot [24, 44]. We used the implementation in version 0.13 of the Rtsne package [25].

The number of significant principal components present in the TCGA transcription factor data set was computed using version 1.1.8 of the PCDimension R package [22]. In order to cluster the set of transcription factors, we used version 0.12.0 of the Thresher R package [19]. The Thresher algorithm combines concepts from principal components analysis, outlier filtering, and von Mises-Fisher mixture models.

We previously conducted extensive simulations to compare our automated extensions to the Bayesian graphical approach of Auer-Gervini, as implemented in the PCDimension package, to other algorithms [22]. We looked at the broken-stick model [45], variants of Bartlett’s test [46], randomization-based procedures introduced by ter Braak [47], and alternative Bayesian approaches [48]. We found that the Auer-Gervini methods were competitive with the most accurate methods overall, and they were two orders of magnitude faster than the ter Braak randomization procedures.

We conducted additional simulations to compare the Thresher algorithm to other clustering algorithms [19]. Specifically, we compared Thresher to all 30 methods implemented in the NbClust R package [49], and to the Simultaneous Clustering and Outlier Detection (SCOD) algorithm [50]. First, we showed that Thresher is consistently more accurate than SCOD at detecting outliers. Second, we found that Thresher clearly had the best performance when there were more variables (or measurements) than there were objects to cluster. (Its performance when there were more objects than variables was good, but not exceptional). The situation with more variables than objects occurs in the most common applications of clustering to omics-scale data, where the number of genes is typically large compared to the number of samples being clustered. In our application of Thresher in this manuscript, we are interested in clustering relatively few objects (486 transcription factors) using a large number of measurements (10,446 patient samples).

Gene enrichment analyses were performed by uploading lists of genes that were highly correlated (|*ρ*|>0.5) to the mean expression vector of each transcription factor to the ToppGene web site [31].

## Additional files

Additional file 1 **Table S0**: Maximum a posteriori estimate of the principal component dimension as a function of the prior parameter, *Θ*. **Tables S2–S15**: Pairwise scatter plots of principle components (1–2, …, 27–28). **Tables S16–S45**: (a) Auer-Gervini plots and (b) scree plots for biological components 1–30. **Tables S46–S75**: Bena plots of biological components 1–30. (PDF 1292 kb)

Additional file 2 Assignments of transcription factors to biological components. (XLSX 35.2 kb)

Additional file 3 Results of ToppGene analysis of each of the thirty biological components. (XLSX 571 kb)

## Abbreviations

AIC: Akaike information criterion
MAP: Maximum a posteriori
PC: Principal component
PCA: principal components analysis TF: Transcription factor
TCGA: The cancer genome atlas
T-SNE: T-distributed stochastic neighbor embedding
