# Supplementary material for: Thirty biologically interpretable clusters of transcription factors distinguish cancer type
Source: BMC Genomics. 2018 Oct 11;19:738. doi: 10.1186/s12864-018-5093-z (PMC6180590; doi:10.1186/s12864-018-5093-z)
Supplement: Supplementary file 1 — Table S0: Maximum a posteriori estimate of the principal component dimension as a function of the prior parameter, Θ. Tables S2–S15: Pairwise scatter plots of principle components (1–2, …, 27–28). Tables S16–S45: (a) Auer-Gervini plots and (b) scree plots for biological components 1–30. Tables S46–S75: Bena plots of biological components 1–30. (PDF 1292 kb) [file 12864_2018_5093_MOESM1_ESM.pdf]

Supplementary Figures

Zachary B. Abrams, Mark Zucker, Min Wang, and Kevin R. Coombes

March 20, 2018

Contents

1 Auer-Gervini Plot **3**

2 Principal Component Plots **3**

3 Auer-Gervini Plots by Biological Component **11**

4 Bean Plots Showing Cancer Type by Biological Component **22**

5 Supplementary Tables **29**

List of Figures

1 Maximum a posteriori estimate of the principal component dimension as a function of the prior parameter, Θ. 3
2 Principal Components 1 and 2. 4
3 Principal Components 3 and 4. 4
4 Principal Components 5 and 6. 5
5 Principal Components 7 and 8. 5
6 Principal Components 9 and 10. 6
7 Principal Components 11 and 12. 6
8 Principal Components 13 and 14. 7
9 Principal Components 15 and 16. 7
10 Principal Components 17 and 18. 8
11 Principal Components 19 and 20. 8
12 Principal Components 21 and 22. 9
13 Principal Components 23 and 24. 9
14 Principal Components 25 and 26. 10
15 Principal Components 27 and 28. 10
16 The genes in biological component 1 form a one-dimensional space. (A) Auer-Gervini plot. (B) Scree plot. 11
17 The genes in biological component 2 form a one-dimensional space. (A) Auer-Gervini plot. (B) Scree plot. 11
18 The genes in biological component 3 form a one-dimensional space. (A) Auer-Gervini plot. (B) Scree plot. 12
19 The genes in biological component 4 form a one-dimensional space. (A) Auer-Gervini plot. (B) Scree plot. 12
20 The genes in biological component 5 form a one-dimensional space. (A) Auer-Gervini plot. (B) Scree plot. 12
21 The genes in biological component 6 form a one-dimensional space. (A) Auer-Gervini plot. (B) Scree plot. 13
22 The genes in biological component 7 form a one-dimensional space. (A) Auer-Gervini plot. (B) Scree plot. 13
23 The genes in biological component 8 form a one-dimensional space. (A) Auer-Gervini plot. (B) Scree plot. 13
24 The genes in biological component 9 form a one-dimensional space. (A) Auer-Gervini plot. (B) Scree plot. 14
25 The genes in biological component 10 form a one-dimensional space. (A) Auer-Gervini plot. (B) Scree plot. 14
26 The genes in biological component 11 form a one-dimensional space. (A) Auer-Gervini plot. (B) Scree plot. 14
27 The genes in biological component 12 form a one-dimensional space. (A) Auer-Gervini plot. (B) Scree plot. 15
28 The genes in biological component 13 form a one-dimensional space. (A) Auer-Gervini plot. (B) Scree plot. 15
29 The genes in biological component 14 form a one-dimensional space. (A) Auer-Gervini plot. (B) Scree plot. 15
30 The genes in biological component 15 form a one-dimensional space. (A) Auer-Gervini plot. (B) Scree plot. 16

31 The genes in biological component 16 form a one-dimensional space. (A) Auer-Gervini plot. (B) Scree plot. 16
32 The genes in biological component 17 form a one-dimensional space. (A) Auer-Gervini plot. (B) Scree plot. 16
33 The genes in biological component 18 form a one-dimensional space. (A) Auer-Gervini plot. (B) Scree plot. 17
34 The genes in biological component 19 form a one-dimensional space. (A) Auer-Gervini plot. (B) Scree plot. 17
35 The genes in biological component 20 form a one-dimensional space. (A) Auer-Gervini plot. (B) Scree plot. 17
36 The genes in biological component 21 form a one-dimensional space. (A) Auer-Gervini plot. (B) Scree plot. 18
37 The genes in biological component 22 form a one-dimensional space. (A) Auer-Gervini plot. (B) Scree plot. 18
38 The genes in biological component 23 form a one-dimensional space. (A) Auer-Gervini plot. (B) Scree plot. 18
39 The genes in biological component 24 form a one-dimensional space. (A) Auer-Gervini plot. (B) Scree plot. 19
40 The genes in biological component 25 form a one-dimensional space. (A) Auer-Gervini plot. (B) Scree plot. 19
41 The genes in biological component 26 form a one-dimensional space. (A) Auer-Gervini plot. (B) Scree plot. 19
42 The genes in biological component 27 form a one-dimensional space. (A) Auer-Gervini plot. (B) Scree plot. 20
43 The genes in biological component 28 form a one-dimensional space. (A) Auer-Gervini plot. (B) Scree plot. 20
44 The genes in biological component 29 form a one-dimensional space. (A) Auer-Gervini plot. (B) Scree plot. 20
45 The genes in biological component 30 form a one-dimensional space. (A) Auer-Gervini plot. (B) Scree plot. 21
46 Bean plot of cancer types for biological component 1. 22
47 Bean plot of cancer types for biological component 2. 22
48 Bean plot of cancer types for biological component 3. 22
49 Bean plot of cancer types for biological component 4. 23
50 Bean plot of cancer types for biological component 5. 23
51 Bean plot of cancer types for biological component 6. 23
52 Bean plot of cancer types for biological component 7. 23
53 Bean plot of cancer types for biological component 8. 23
54 Bean plot of cancer types for biological component 9. 24
55 Bean plot of cancer types for biological component 10. 24
56 Bean plot of cancer types for biological component 11. 24
57 Bean plot of cancer types for biological component 12. 24
58 Bean plot of cancer types for biological component 13. 24
59 Bean plot of cancer types for biological component 14. 25
60 Bean plot of cancer types for biological component 15. 25
61 Bean plot of cancer types for biological component 16. 25
62 Bean plot of cancer types for biological component 17. 25
63 Bean plot of cancer types for biological component 18. 25
64 Bean plot of cancer types for biological component 19. 26
65 Bean plot of cancer types for biological component 20. 26
66 Bean plot of cancer types for biological component 21. 26
67 Bean plot of cancer types for biological component 22. 26
68 Bean plot of cancer types for biological component 23. 26
69 Bean plot of cancer types for biological component 24. 27
70 Bean plot of cancer types for biological component 25. 27
71 Bean plot of cancer types for biological component 26. 27
72 Bean plot of cancer types for biological component 27. 27
73 Bean plot of cancer types for biological component 28. 27
74 Bean plot of cancer types for biological component 29. 28
75 Bean plot of cancer types for biological component 30. 28

1 Auer-Gervini Plot

Here is the Uaer-Gervini plot for the complete transcription factor dataset.

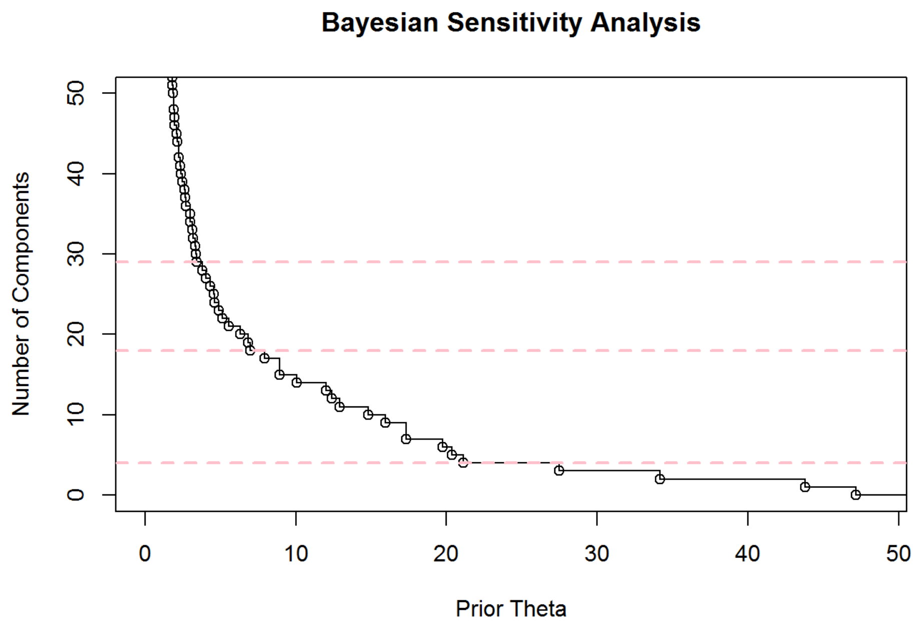

Supplementary Figure 1: Maximum a posteriori estimate of the principal component dimension as a function of the prior parameter, Θ.

2 Principal Component Plots

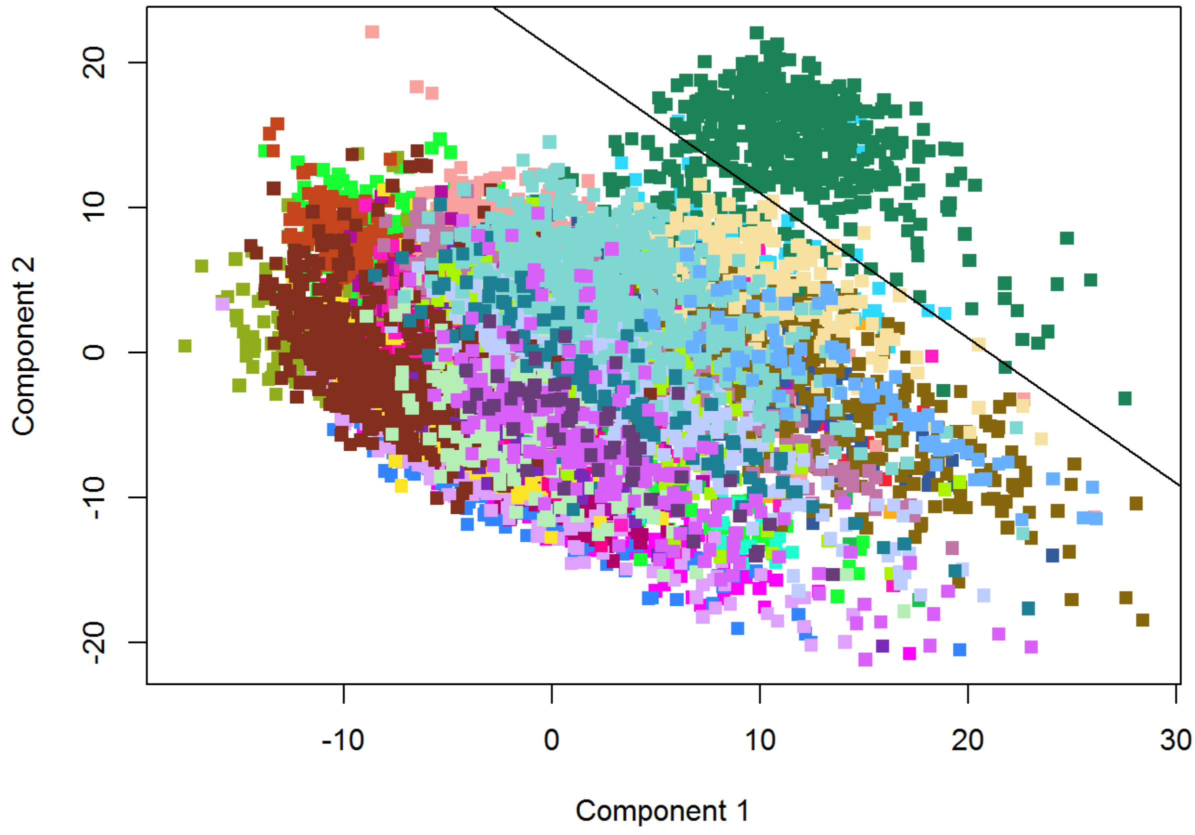

Supplementary Figure 2: Principal Components 1 and 2.

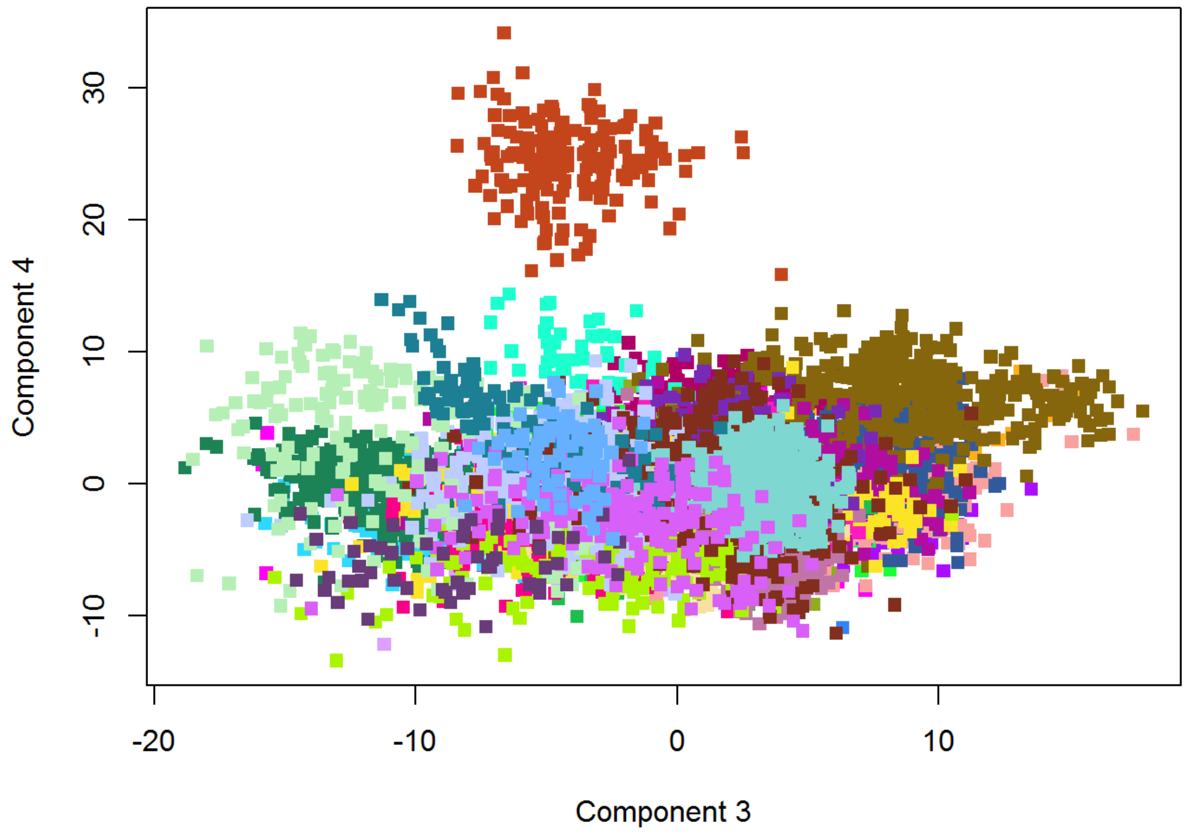

Supplementary Figure 3: Principal Components 3 and 4.

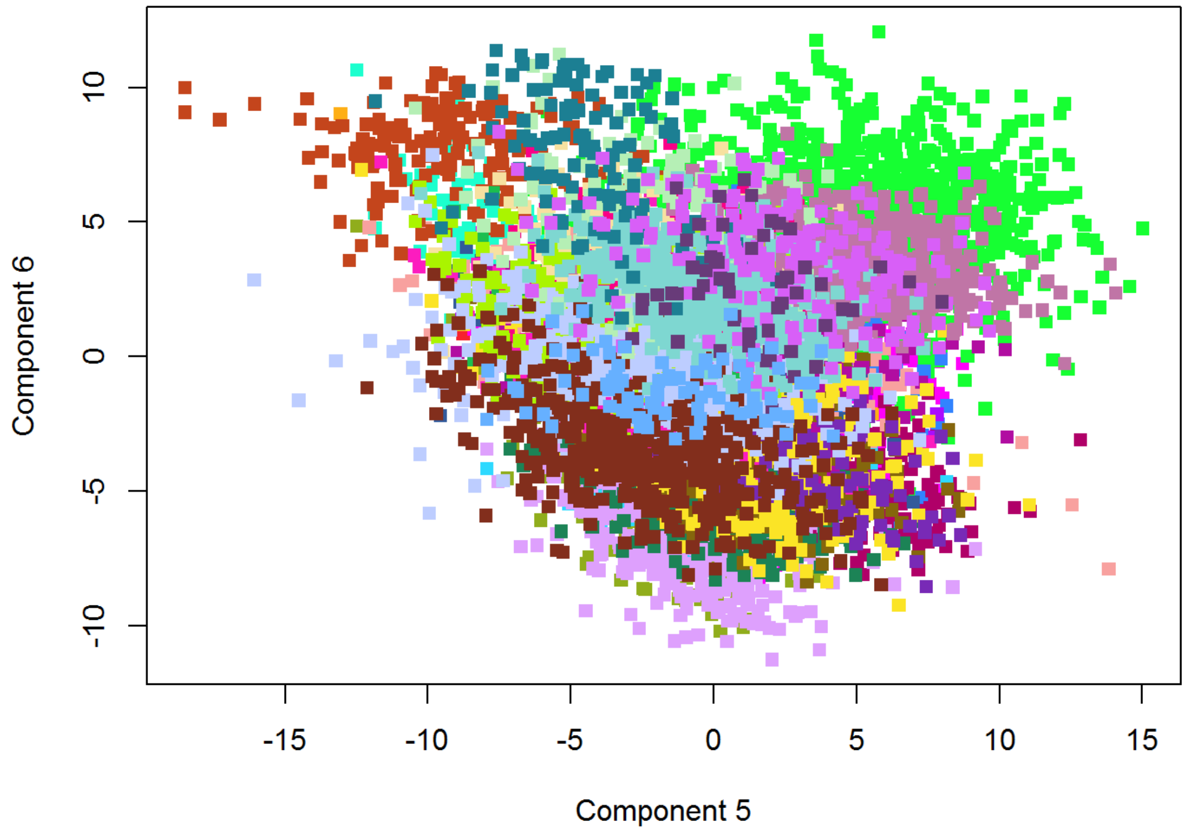

Supplementary Figure 4: Principal Components 5 and 6.

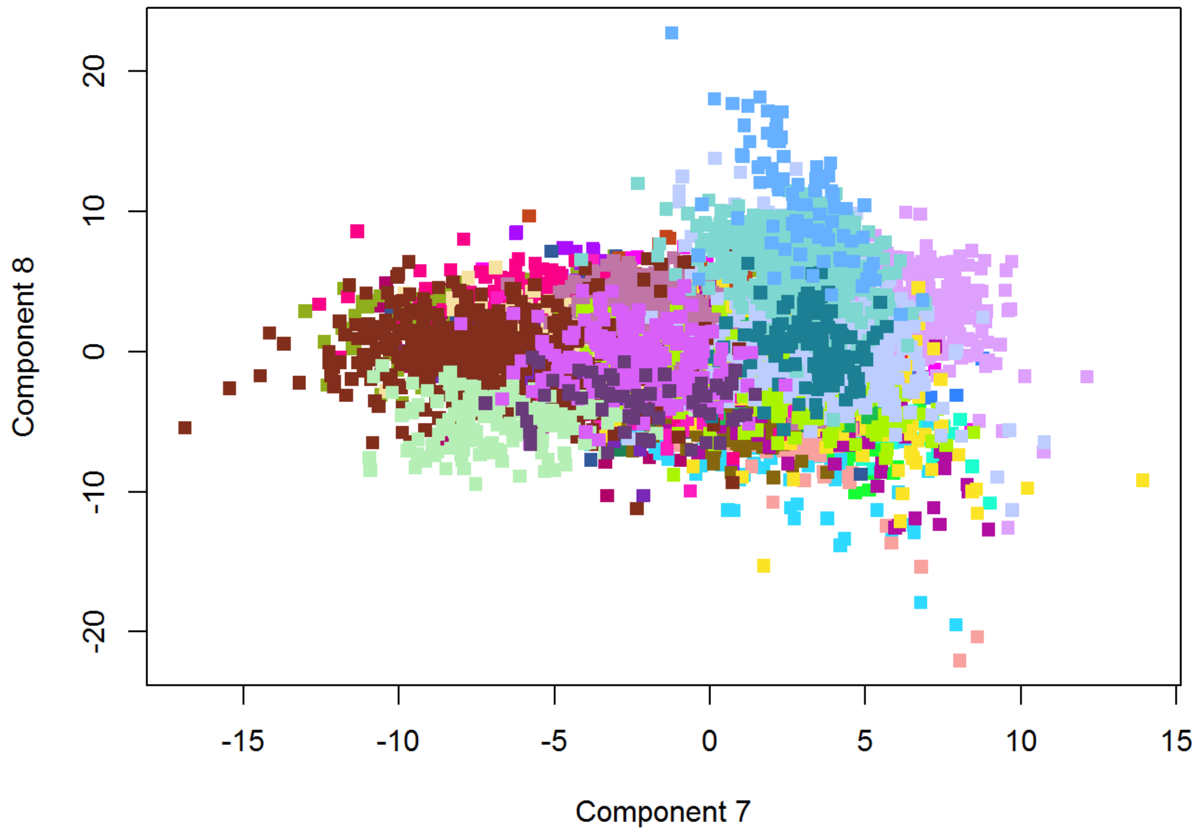

Supplementary Figure 5: Principal Components 7 and 8.

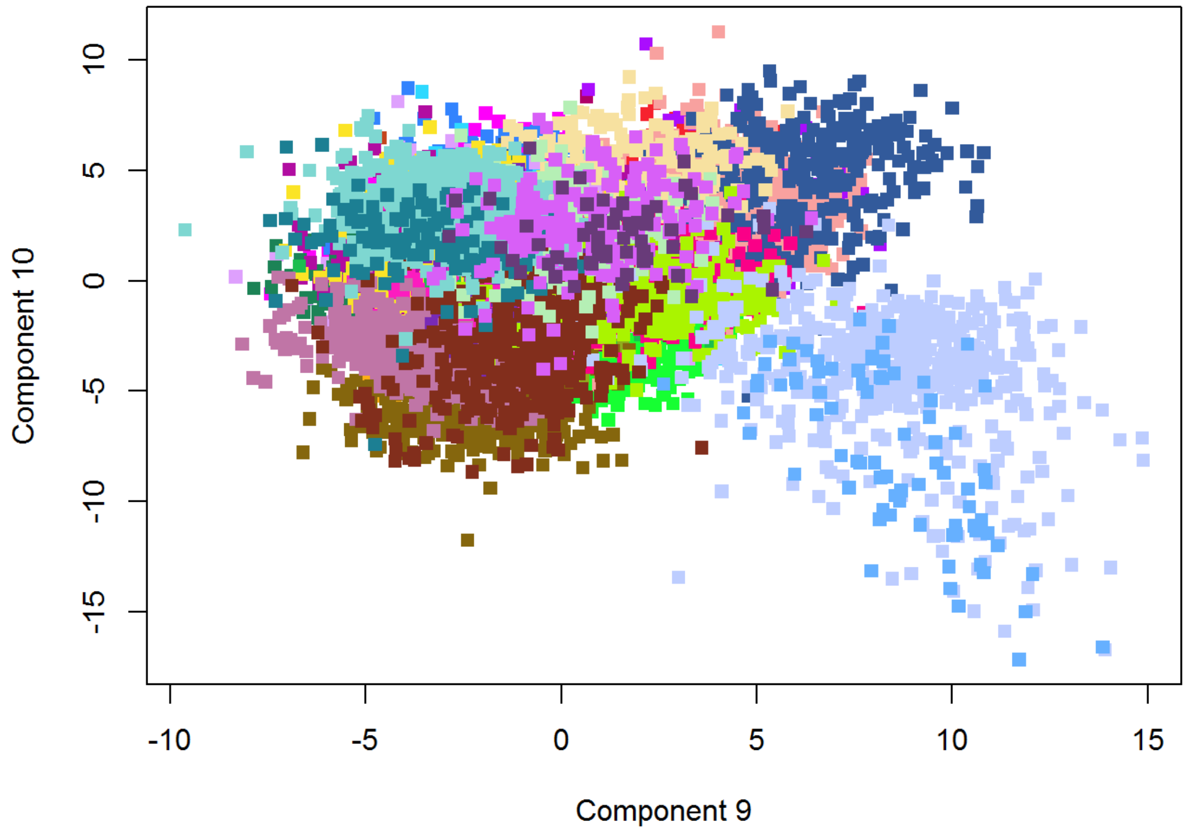

Supplementary Figure 6: Principal Components 9 and 10.

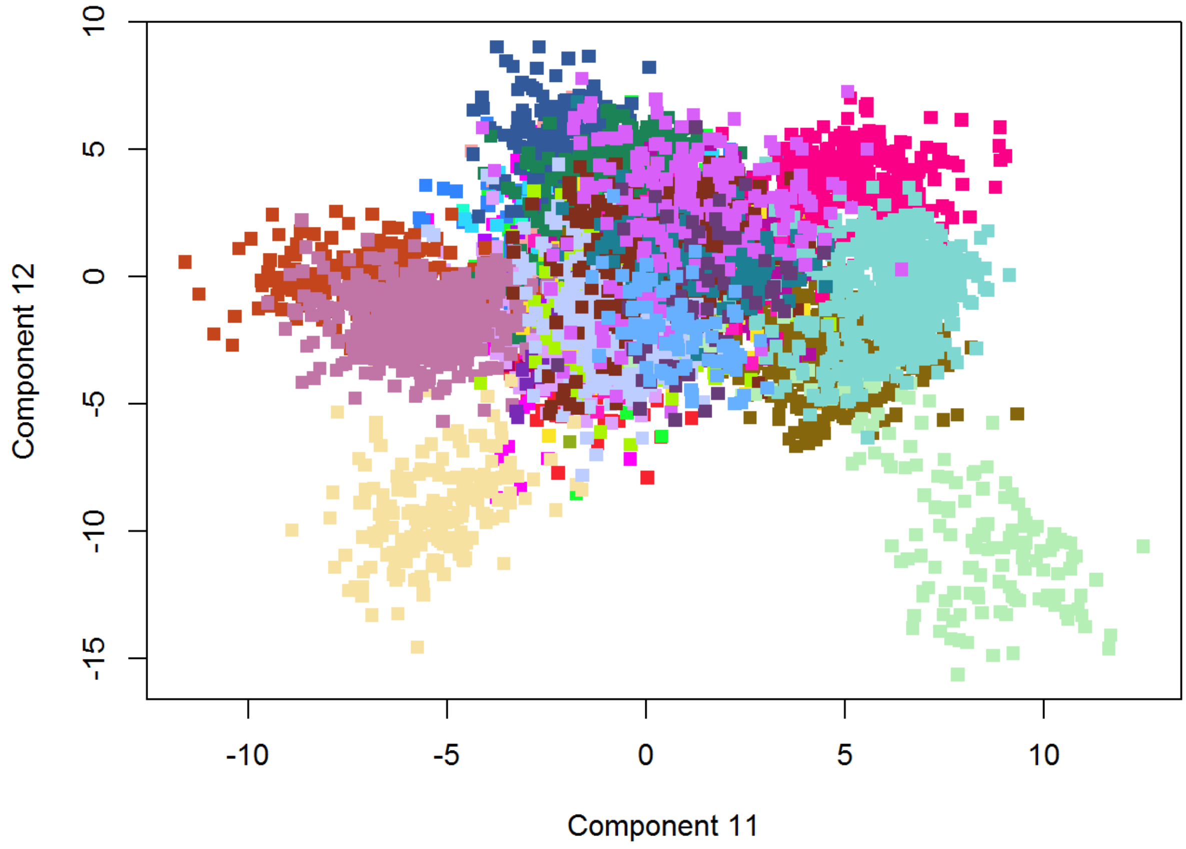

Supplementary Figure 7: Principal Components 11 and 12.

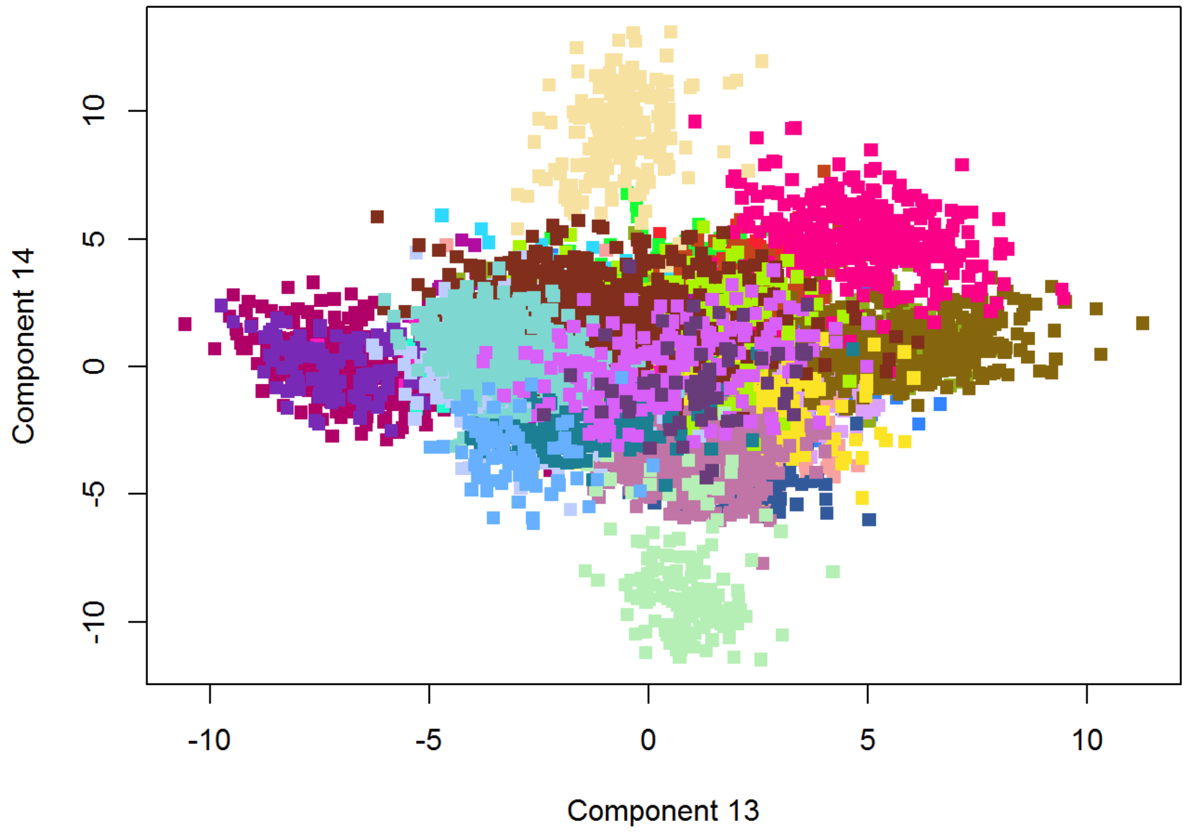

Supplementary Figure 8: Principal Components 13 and 14.

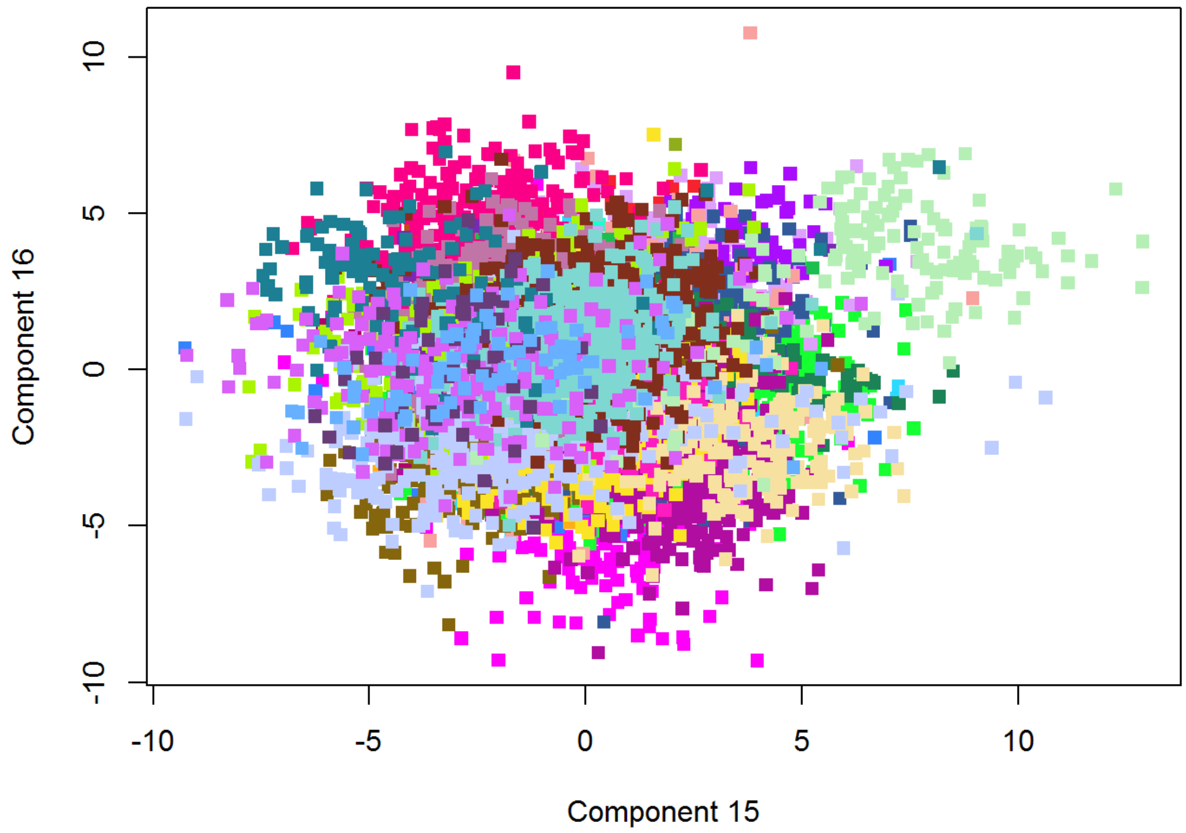

Supplementary Figure 9: Principal Components 15 and 16.

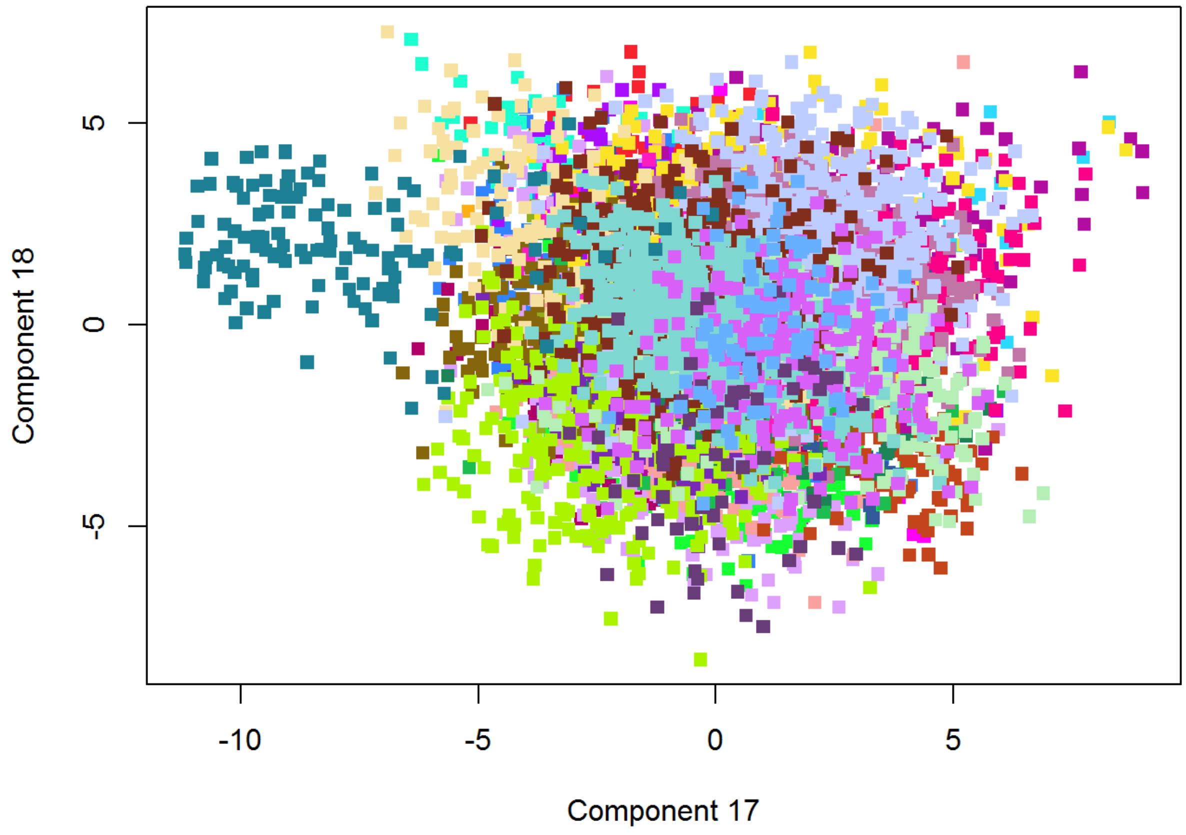

Supplementary Figure 10: Principal Components 17 and 18.

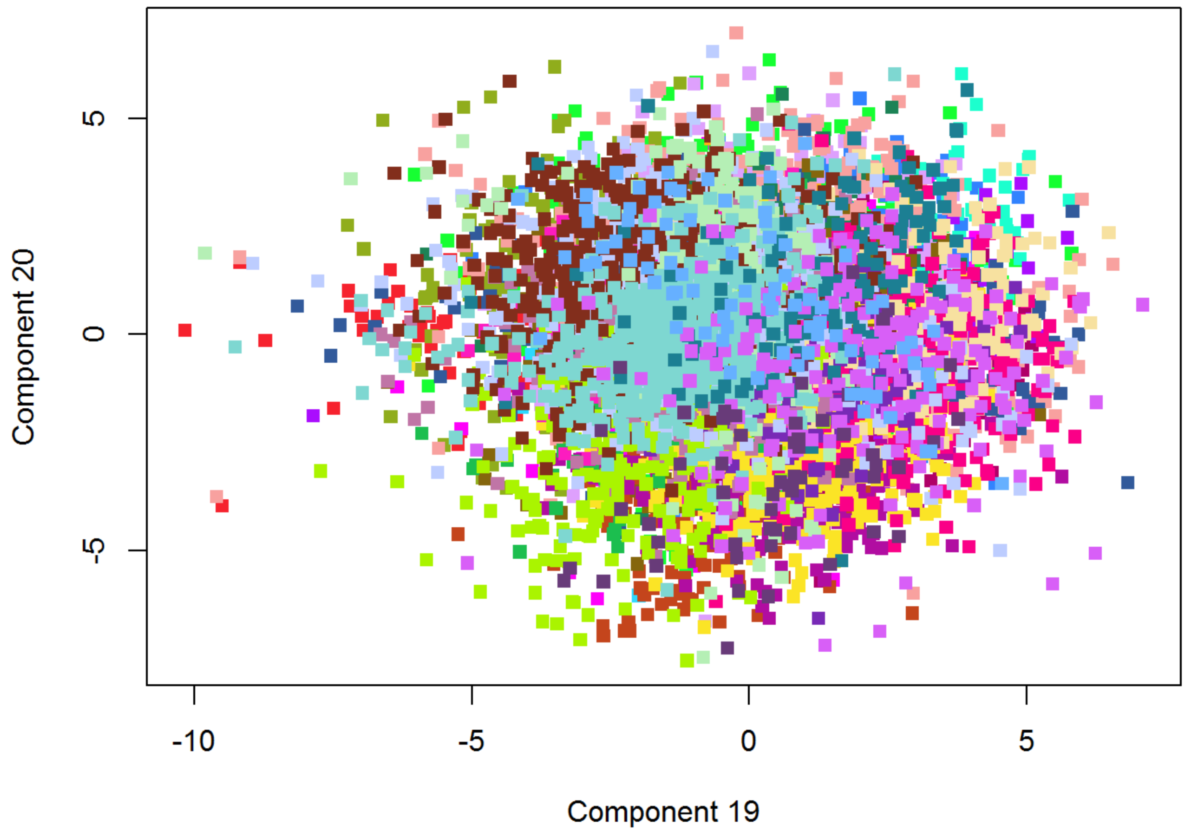

Supplementary Figure 11: Principal Components 19 and 20.

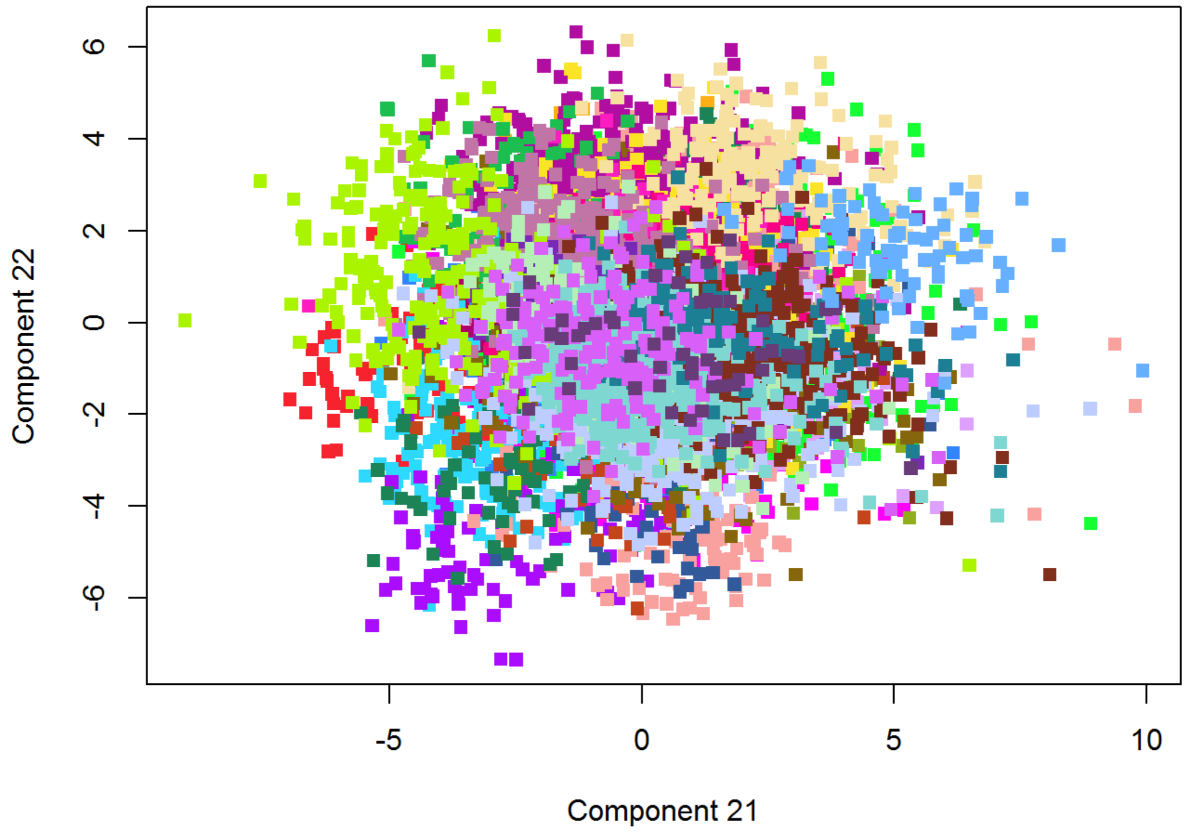

Supplementary Figure 12: Principal Components 21 and 22.

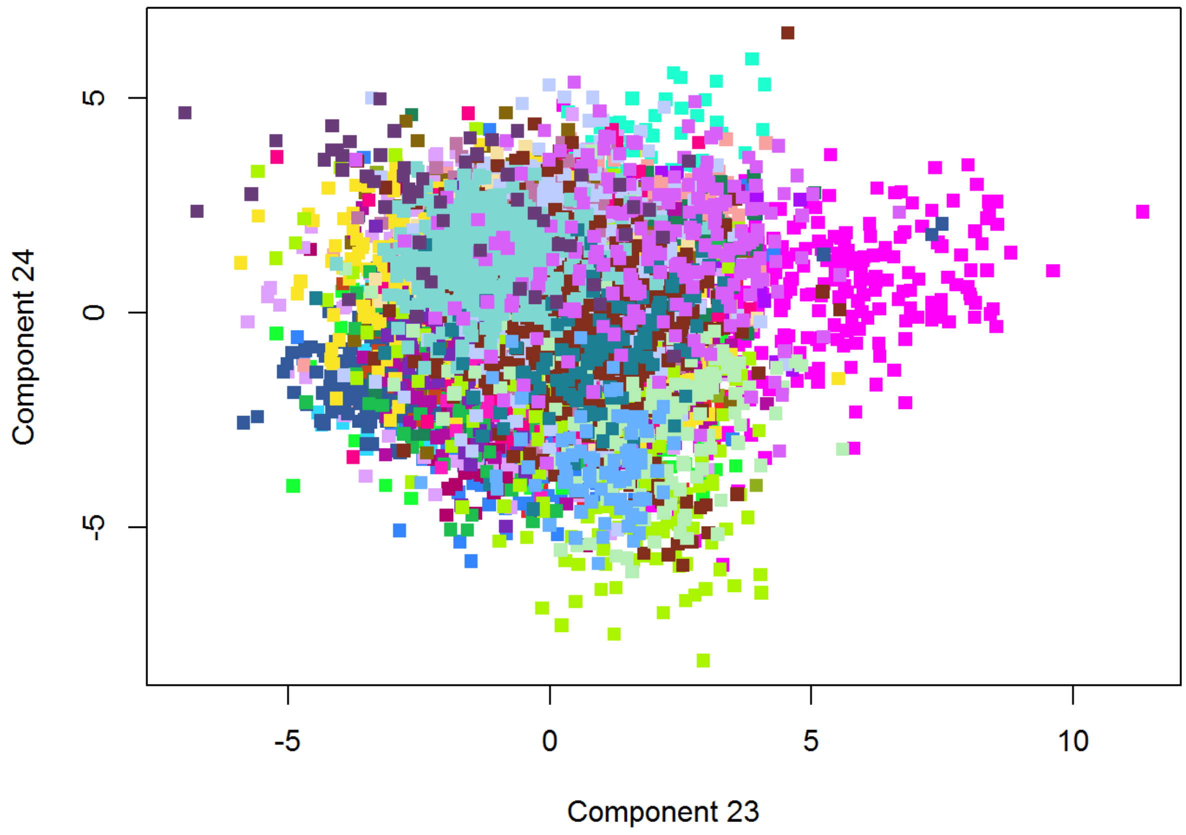

Supplementary Figure 13: Principal Components 23 and 24.

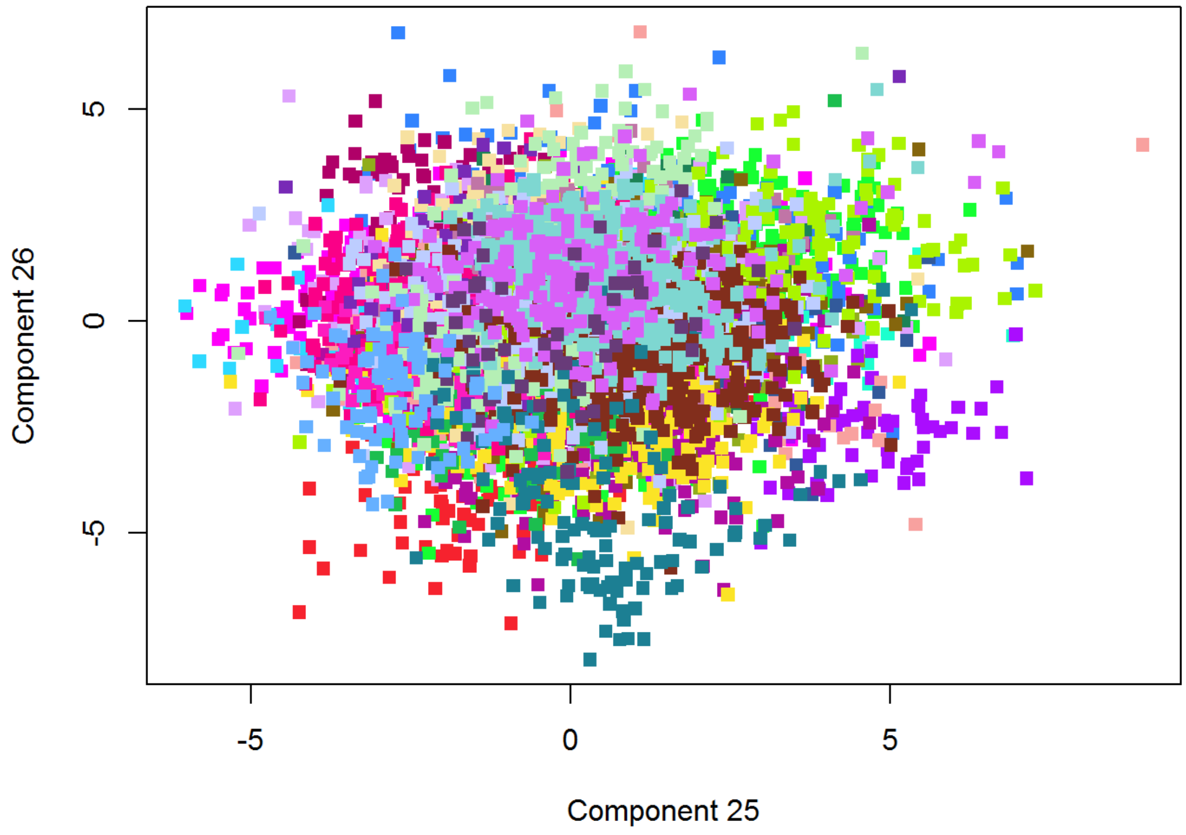

Supplementary Figure 14: Principal Components 25 and 26.

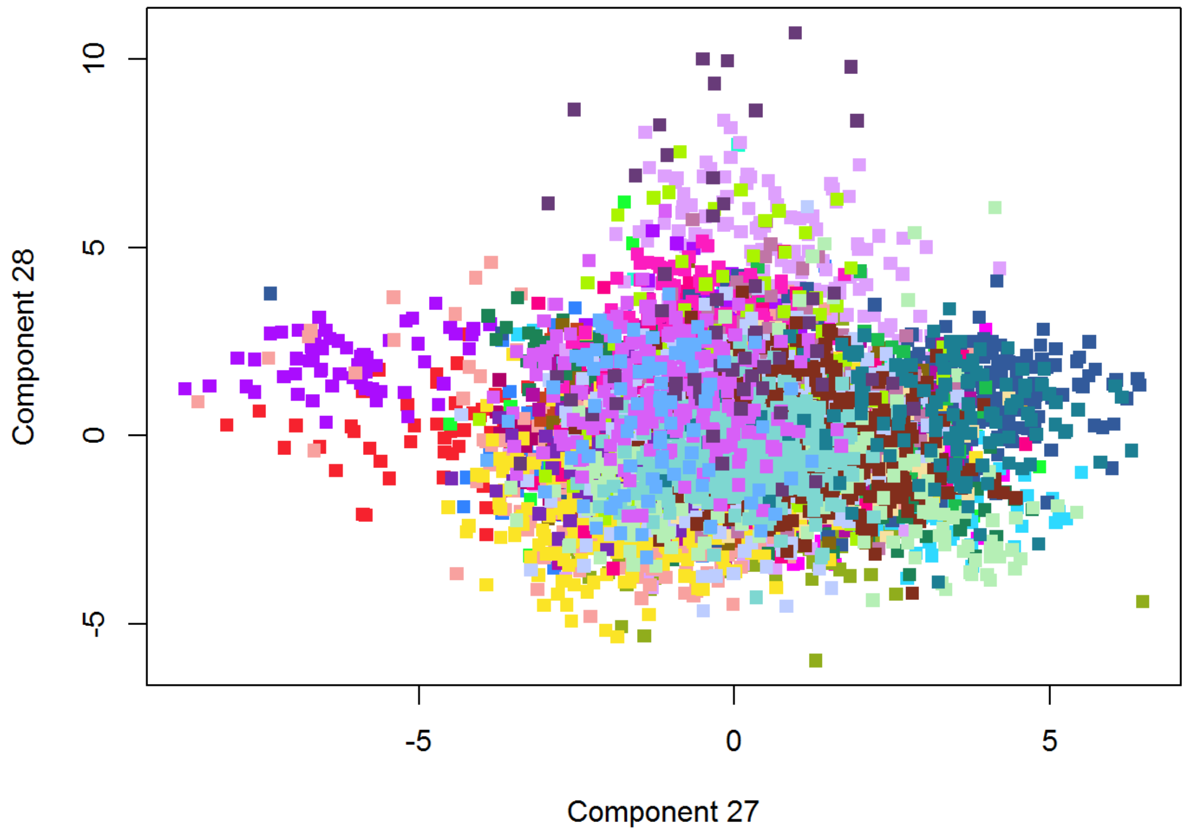

Supplementary Figure 15: Principal Components 27 and 28.

3 Auer-Gervini Plots by Biological Component

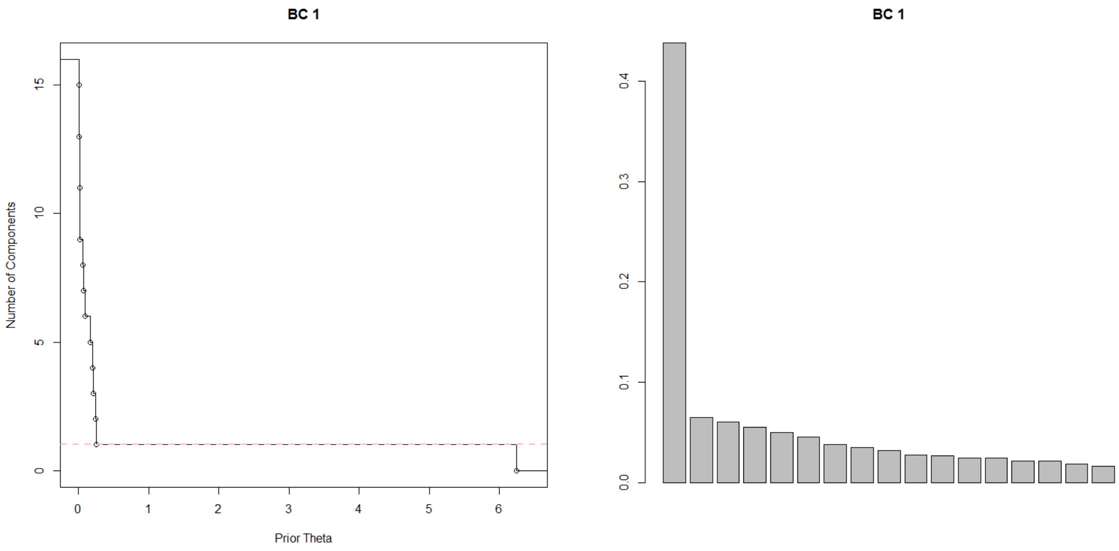

Supplementary Figure 16: The genes in biological component 1 form a one-dimensional space. (A) Auer-Gervini plot. (B) Scree plot.

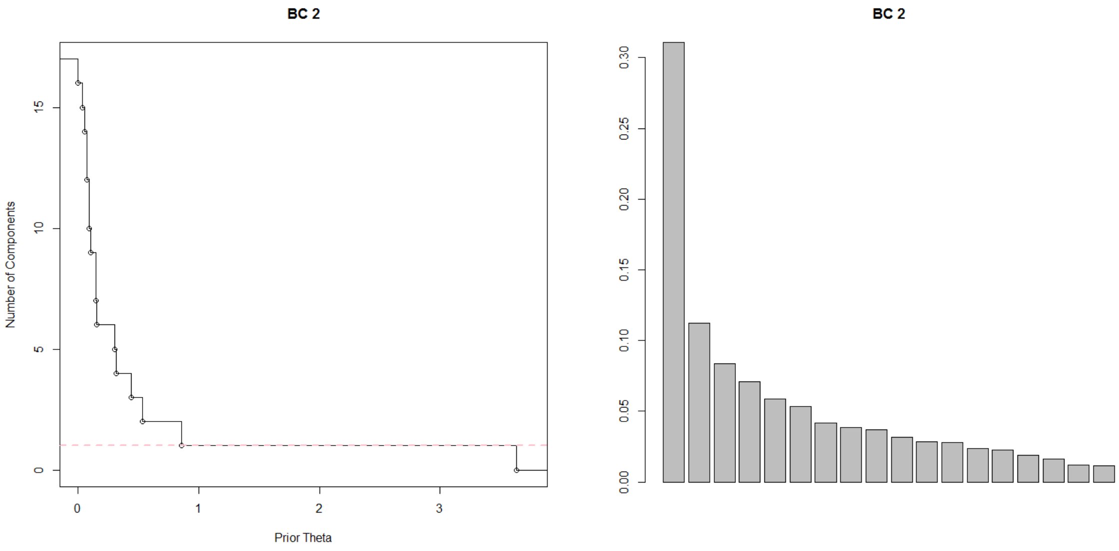

Supplementary Figure 17: The genes in biological component 2 form a one-dimensional space. (A) Auer-Gervini plot. (B) Scree plot.

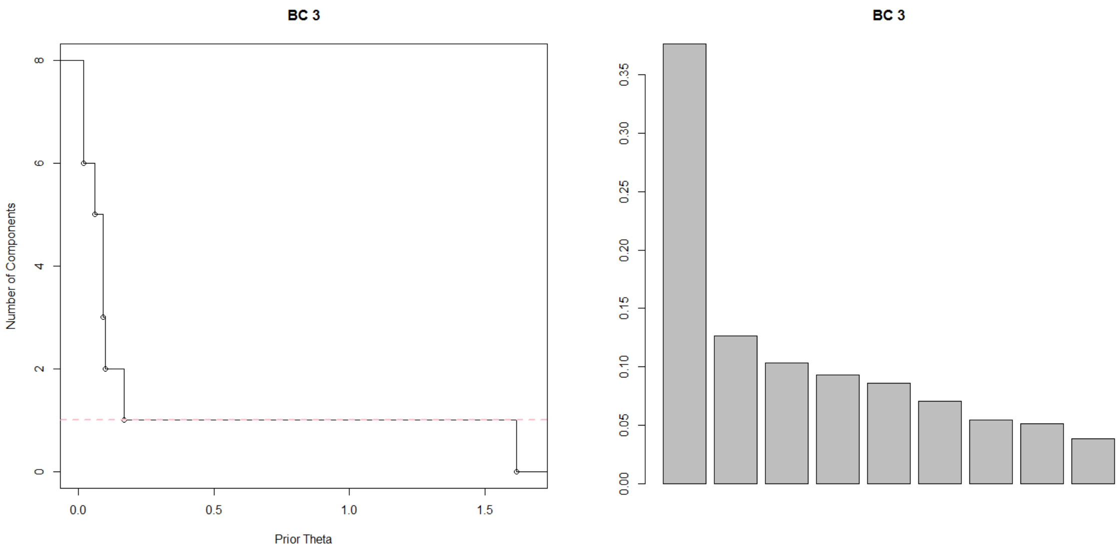

Supplementary Figure 18: The genes in biological component 3 form a one-dimensional space. (A) Auer-Gervini plot. (B) Scree plot.

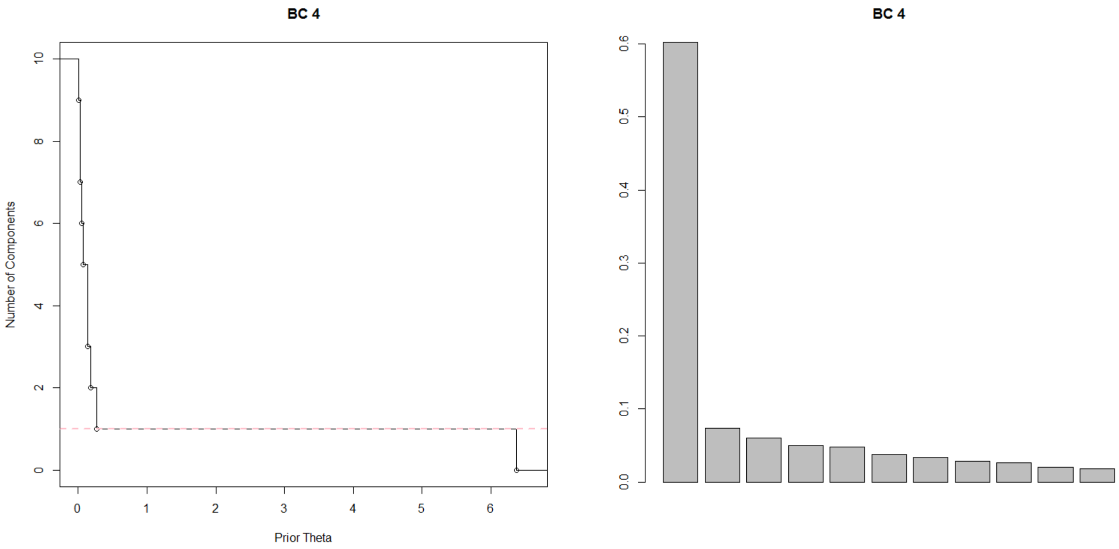

Supplementary Figure 19: The genes in biological component 4 form a one-dimensional space. (A) Auer-Gervini plot. (B) Scree plot.

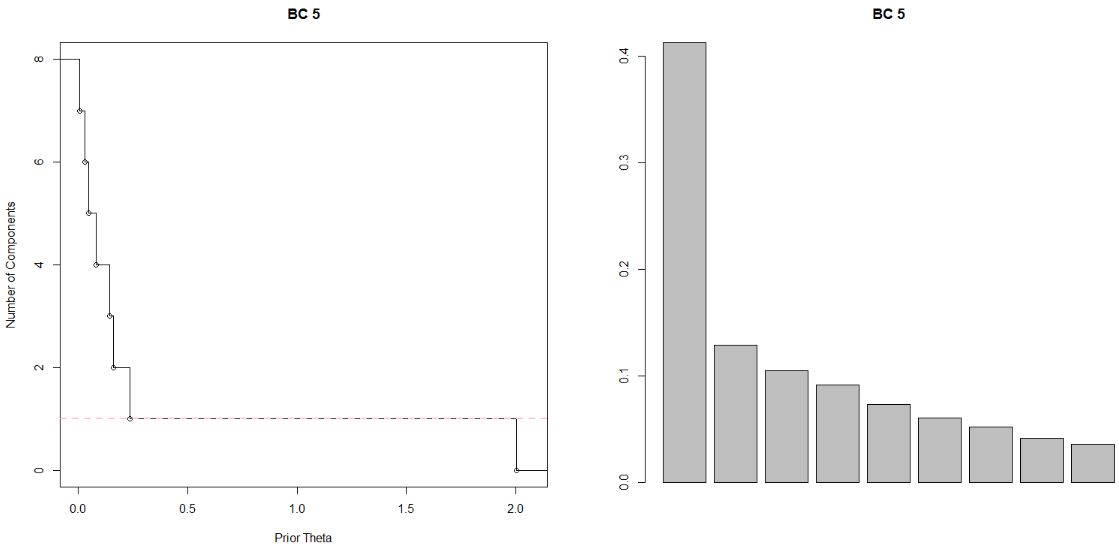

Supplementary Figure 20: The genes in biological component 5 form a one-dimensional space. (A) Auer-Gervini plot. (B) Scree plot.

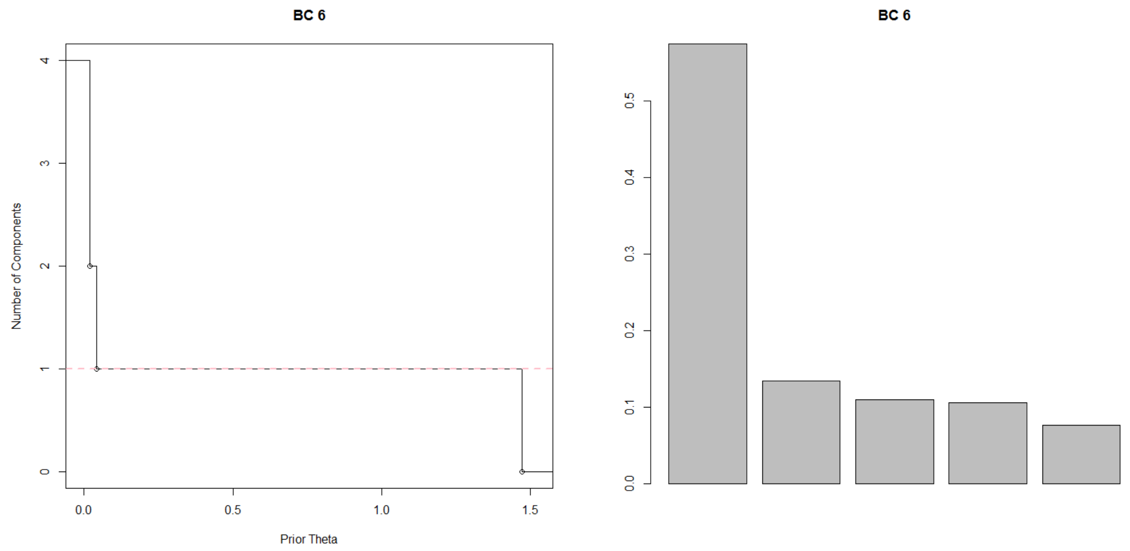

Supplementary Figure 21: The genes in biological component 6 form a one-dimensional space. (A) Auer-Gervini plot. (B) Scree plot.

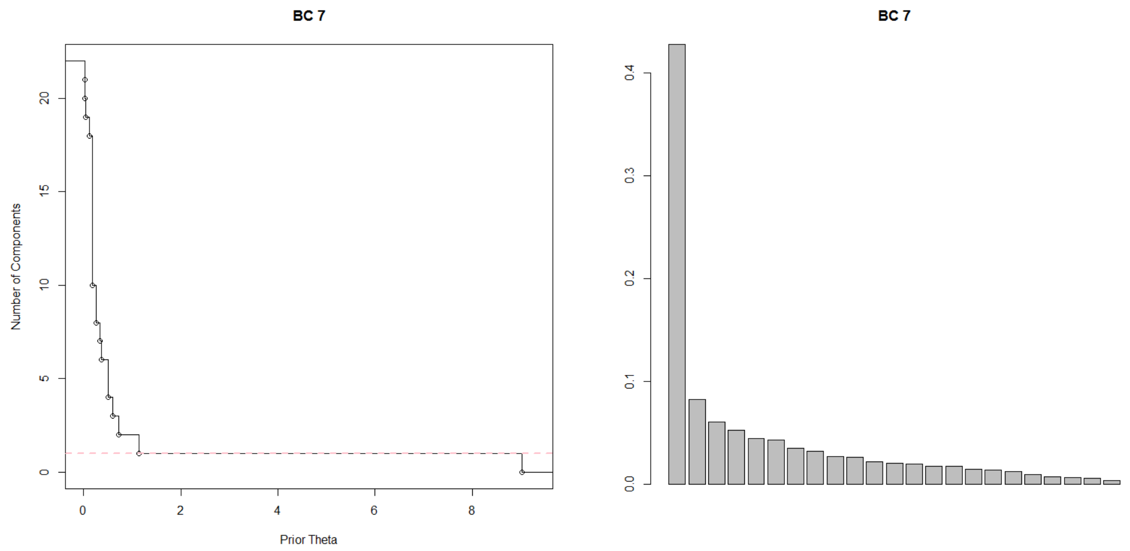

Supplementary Figure 22: The genes in biological component 7 form a one-dimensional space. (A) Auer-Gervini plot. (B) Scree plot.

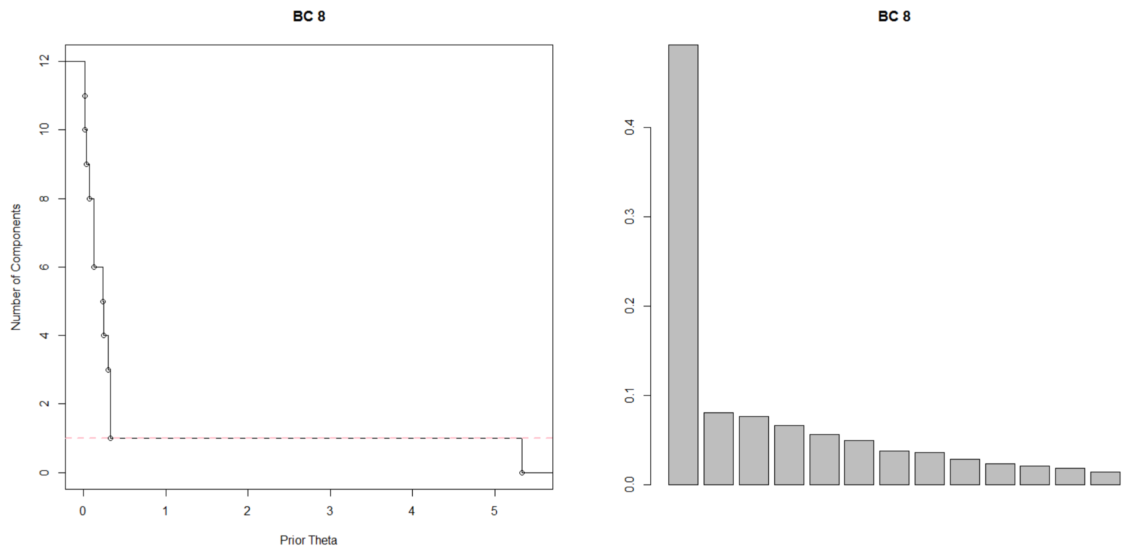

Supplementary Figure 23: The genes in biological component 8 form a one-dimensional space. (A) Auer-Gervini plot. (B) Scree plot.

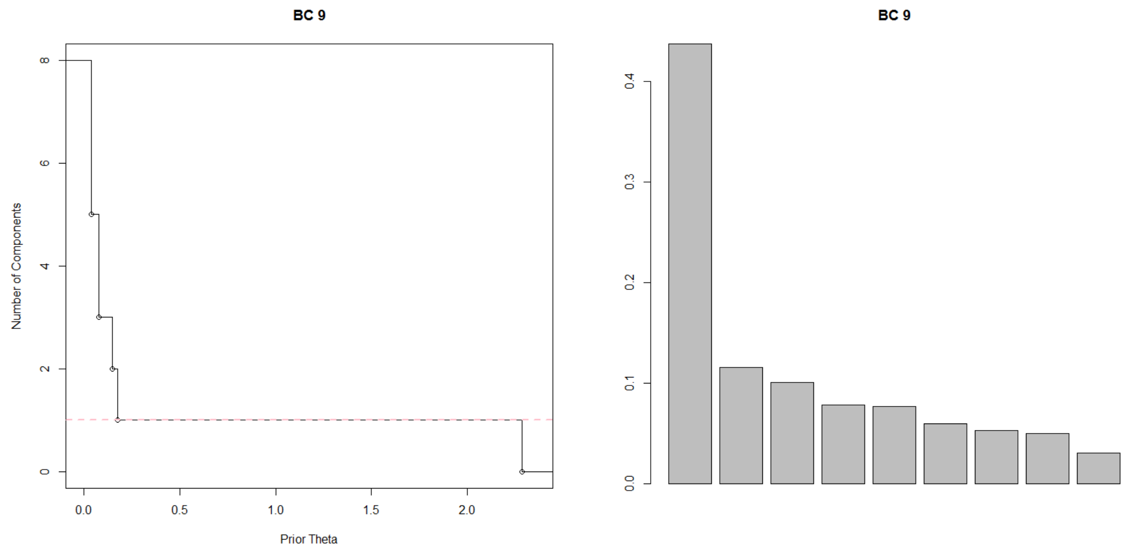

Supplementary Figure 24: The genes in biological component 9 form a one-dimensional space. (A) Auer-Gervini plot. (B) Scree plot.

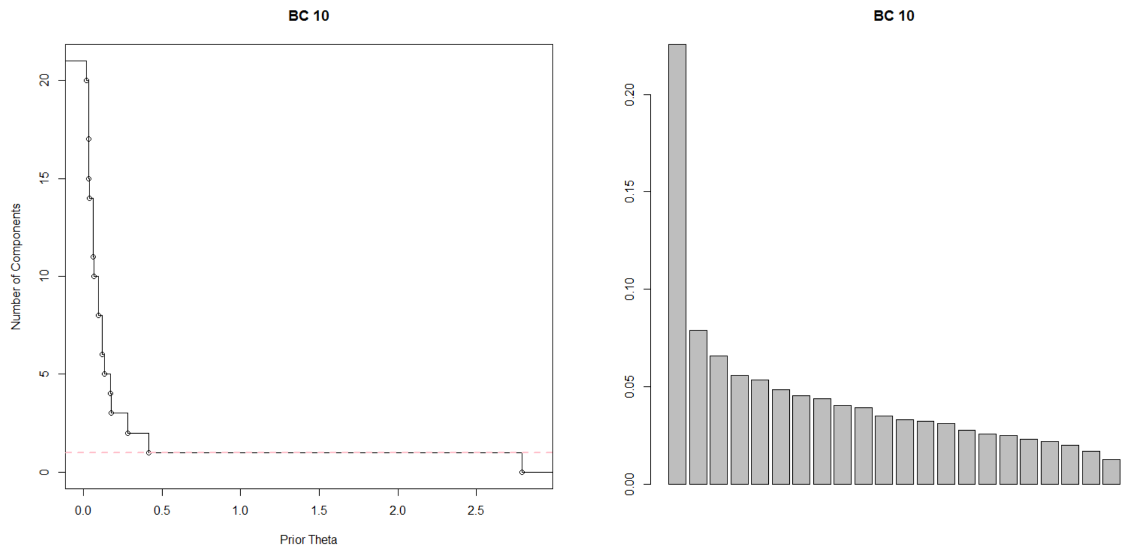

Supplementary Figure 25: The genes in biological component 10 form a one-dimensional space. (A) Auer-Gervini plot. (B) Scree plot.

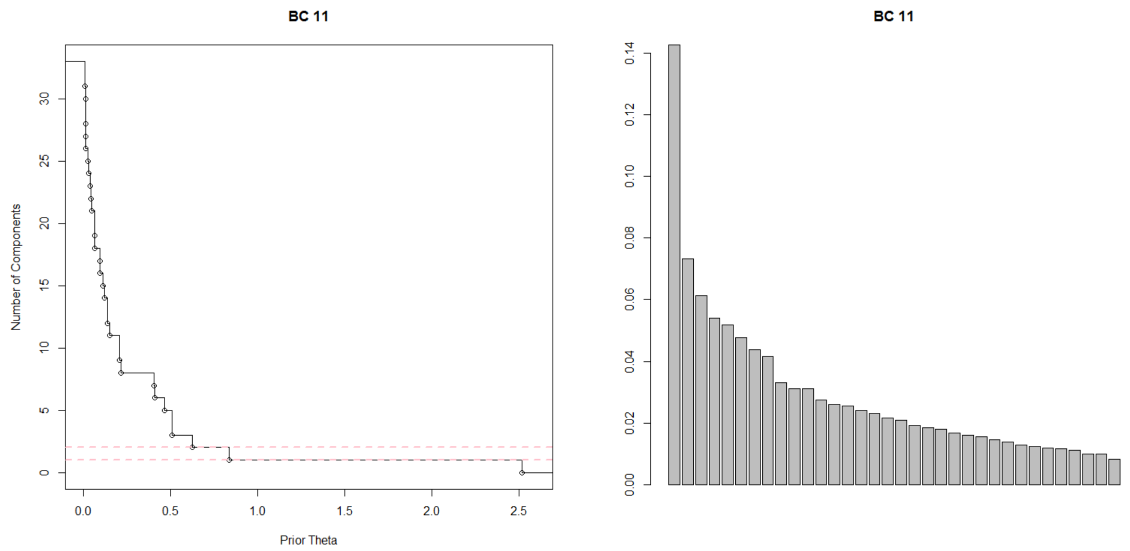

Supplementary Figure 26: The genes in biological component 11 form a one-dimensional space. (A) Auer-Gervini plot. (B) Scree plot.

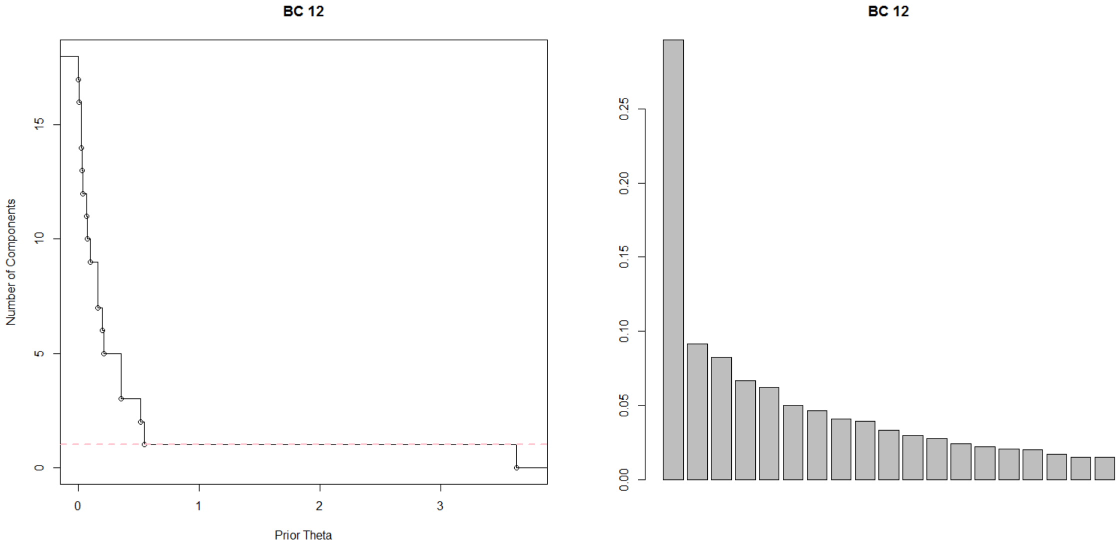

Supplementary Figure 27: The genes in biological component 12 form a one-dimensional space. (A) Auer-Gervini plot. (B) Scree plot.

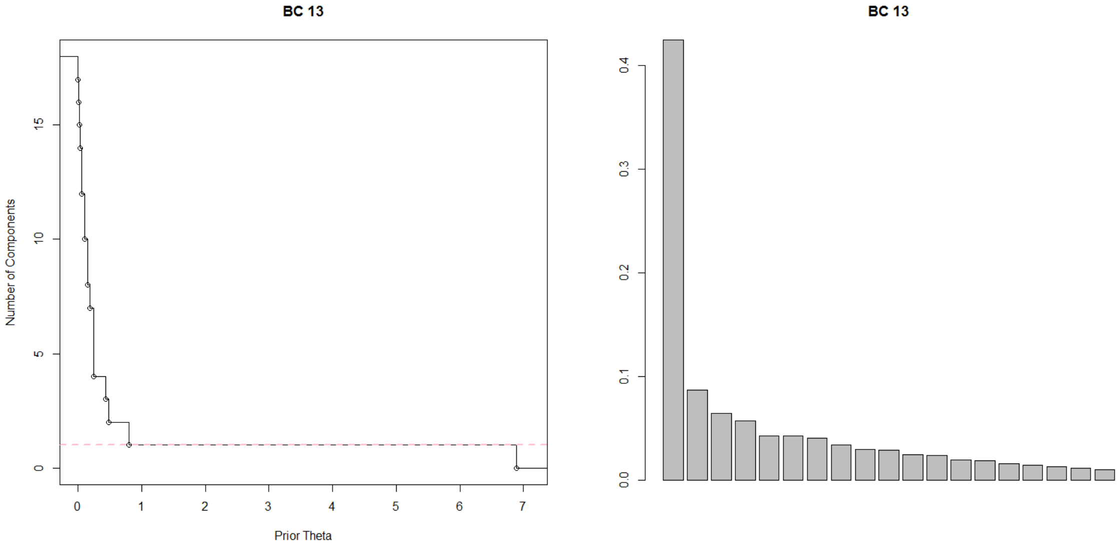

Supplementary Figure 28: The genes in biological component 13 form a one-dimensional space. (A) Auer-Gervini plot. (B) Scree plot.

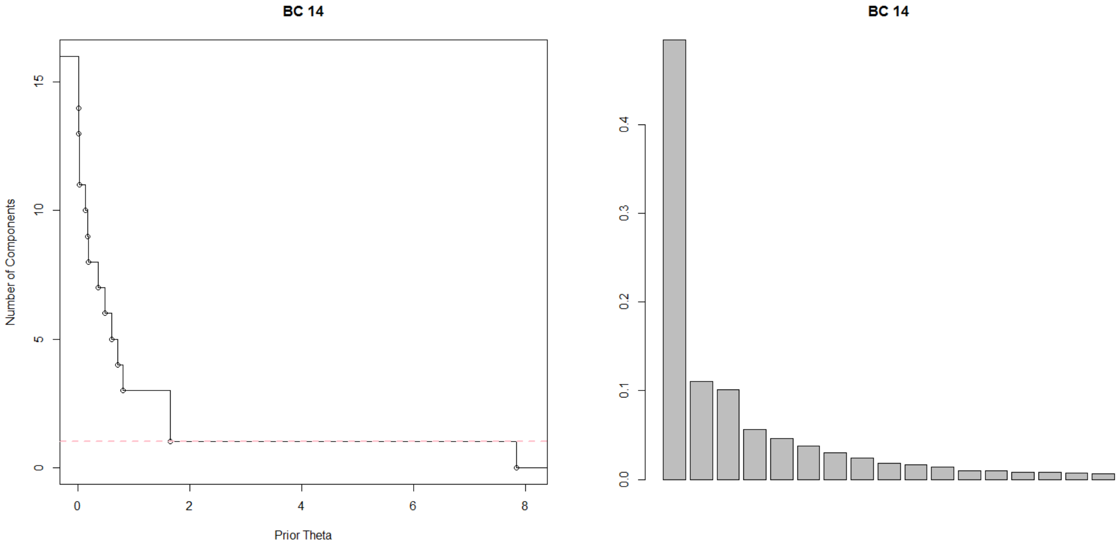

Supplementary Figure 29: The genes in biological component 14 form a one-dimensional space. (A) Auer-Gervini plot. (B) Scree plot.

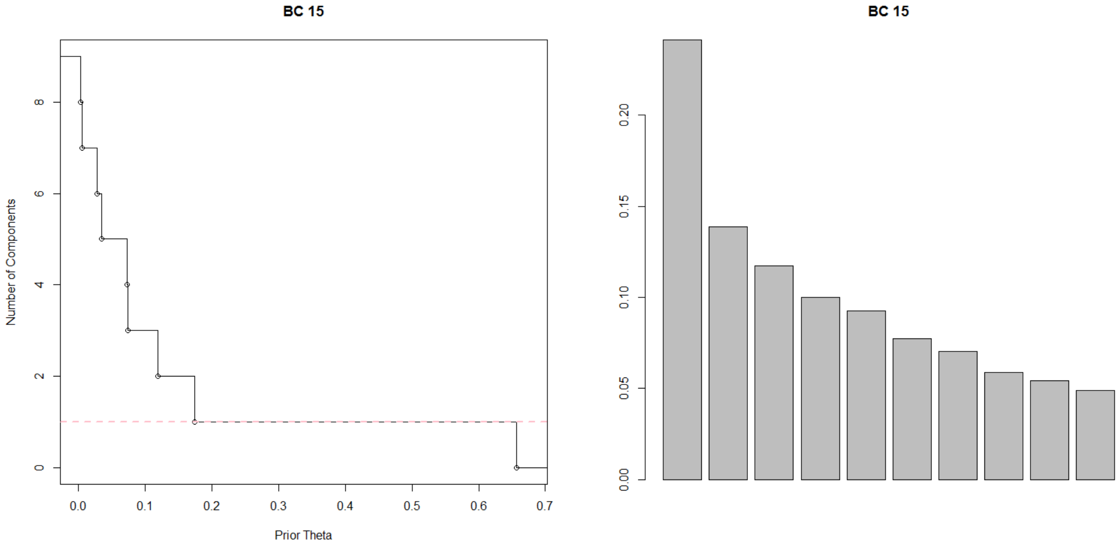

Supplementary Figure 30: The genes in biological component 15 form a one-dimensional space. (A) Auer-Gervini plot. (B) Scree plot.

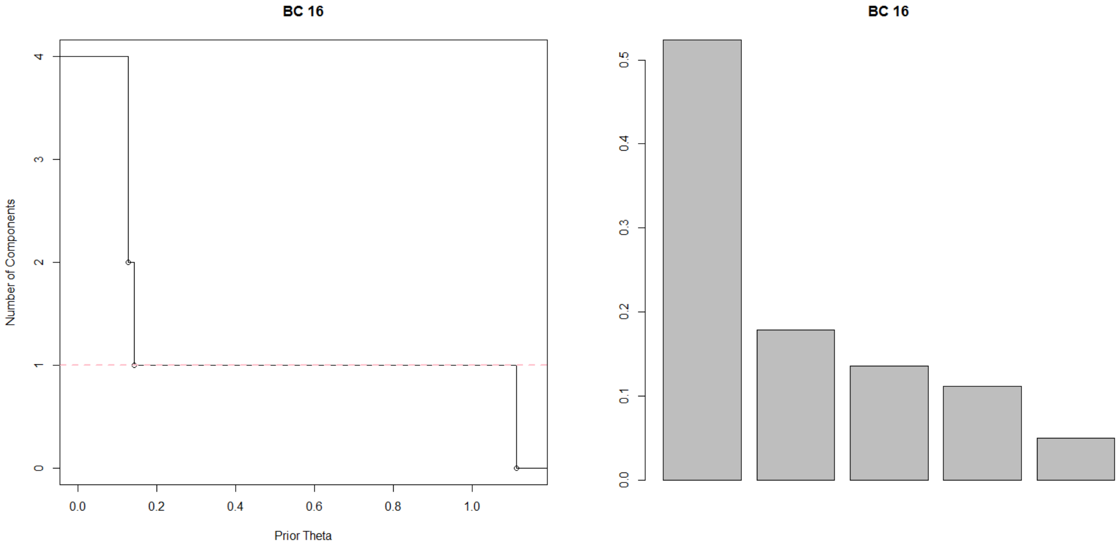

Supplementary Figure 31: The genes in biological component 16 form a one-dimensional space. (A) Auer-Gervini plot. (B) Scree plot.

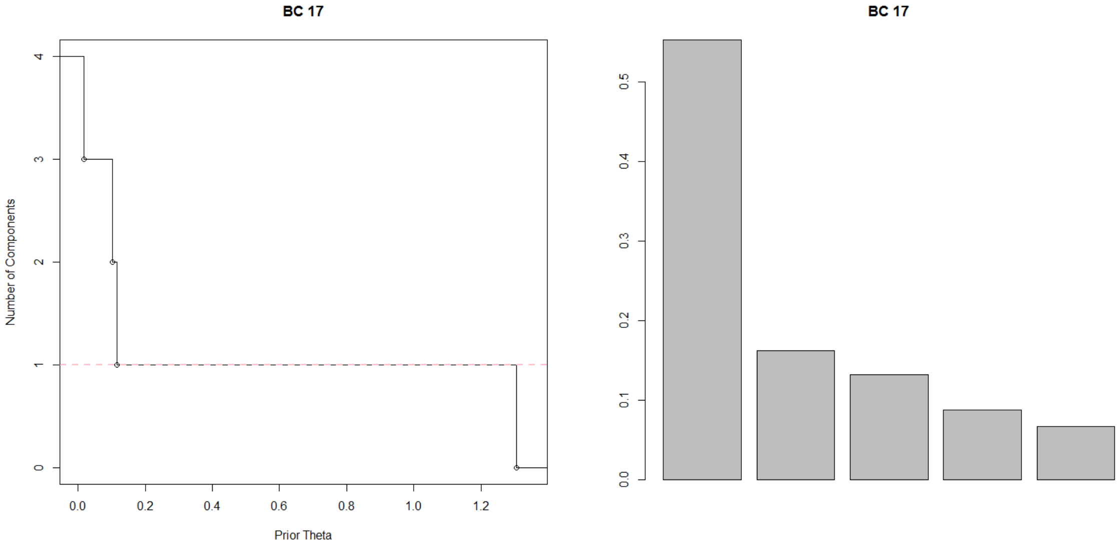

Supplementary Figure 32: The genes in biological component 17 form a one-dimensional space. (A) Auer-Gervini plot. (B) Scree plot.

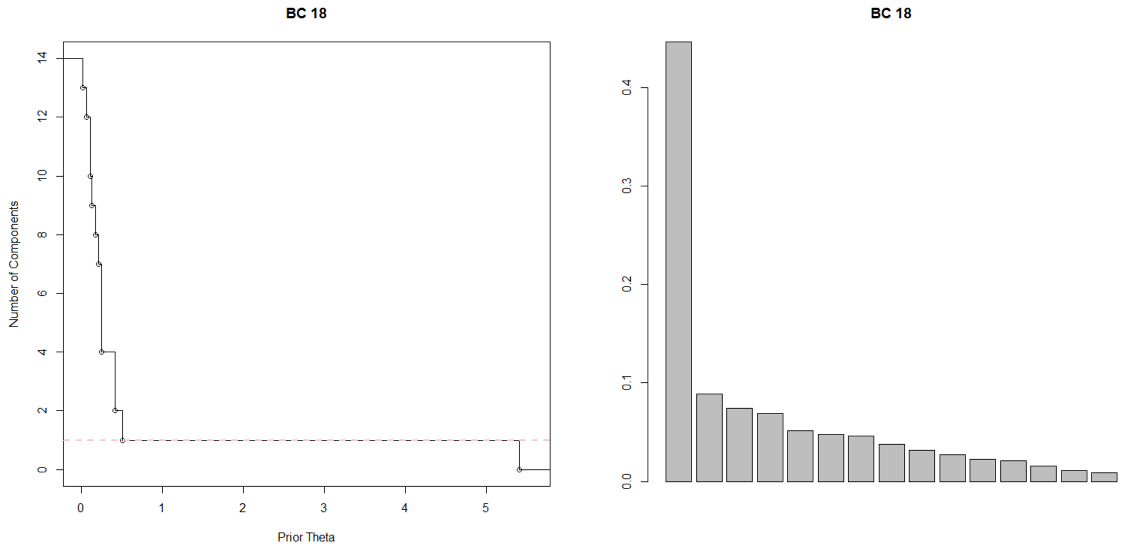

Supplementary Figure 33: The genes in biological component 18 form a one-dimensional space. (A) Auer-Gervini plot. (B) Scree plot.

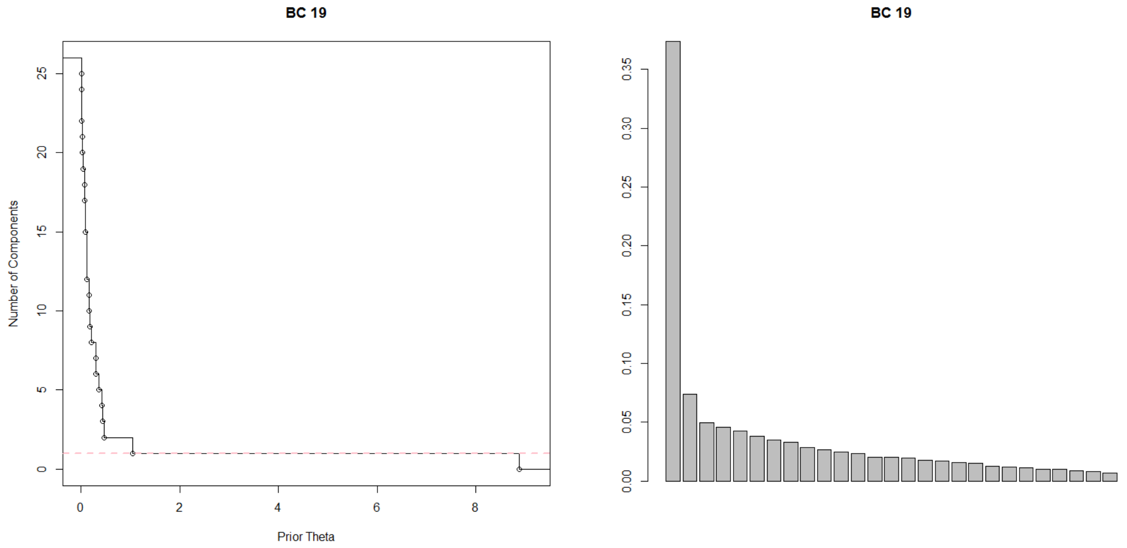

Supplementary Figure 34: The genes in biological component 19 form a one-dimensional space. (A) Auer-Gervini plot. (B) Scree plot.

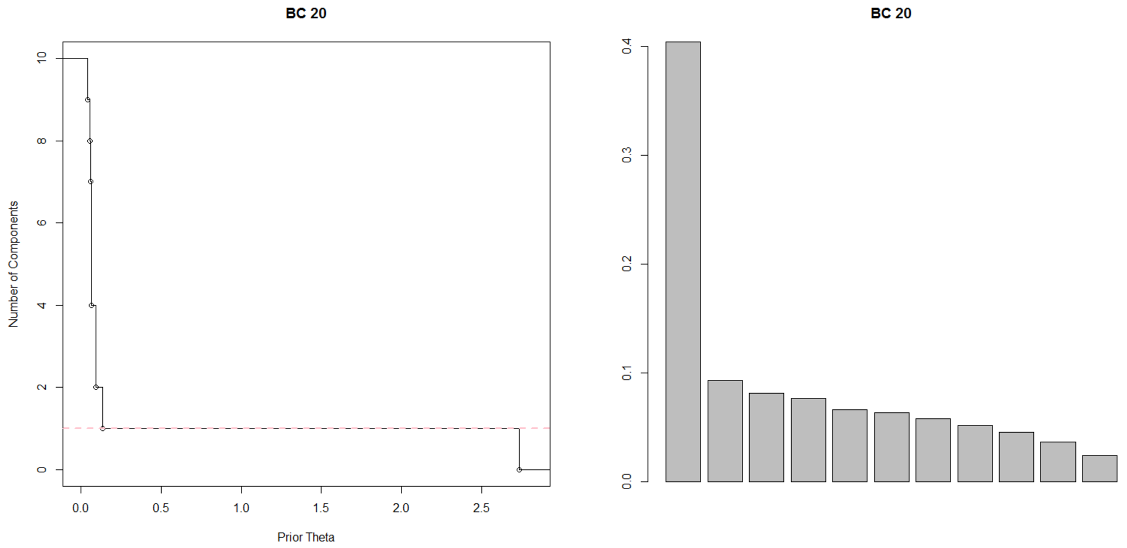

Supplementary Figure 35: The genes in biological component 20 form a one-dimensional space. (A) Auer-Gervini plot. (B) Scree plot.

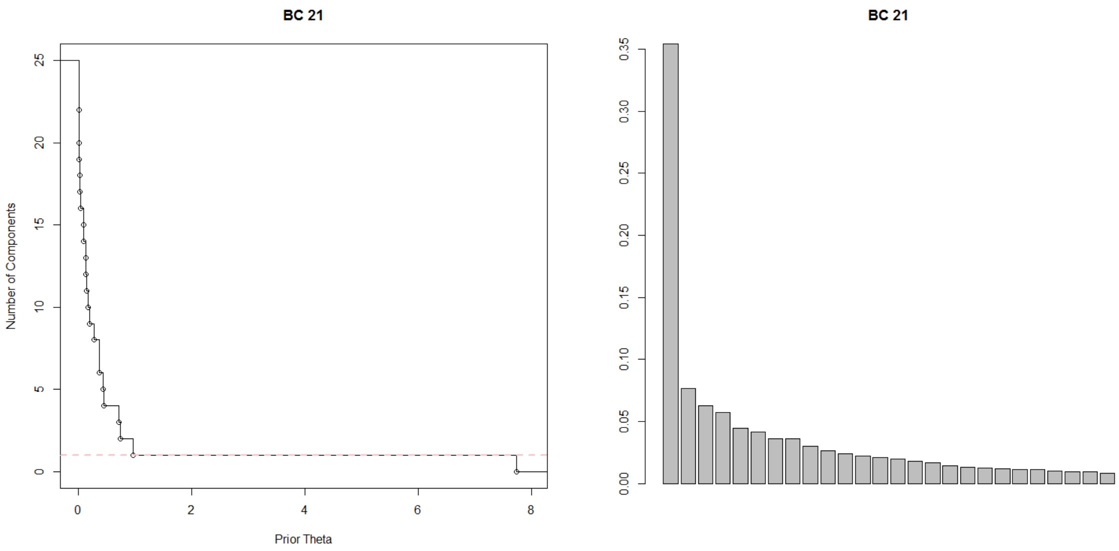

Supplementary Figure 36: The genes in biological component 21 form a one-dimensional space. (A) Auer-Gervini plot. (B) Scree plot.

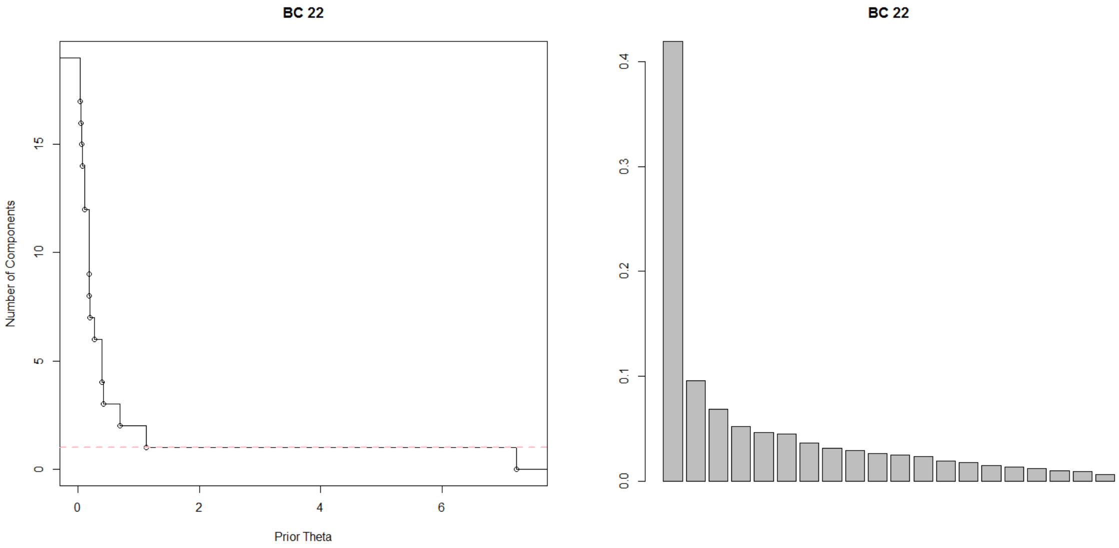

Supplementary Figure 37: The genes in biological component 22 form a one-dimensional space. (A) Auer-Gervini plot. (B) Scree plot.

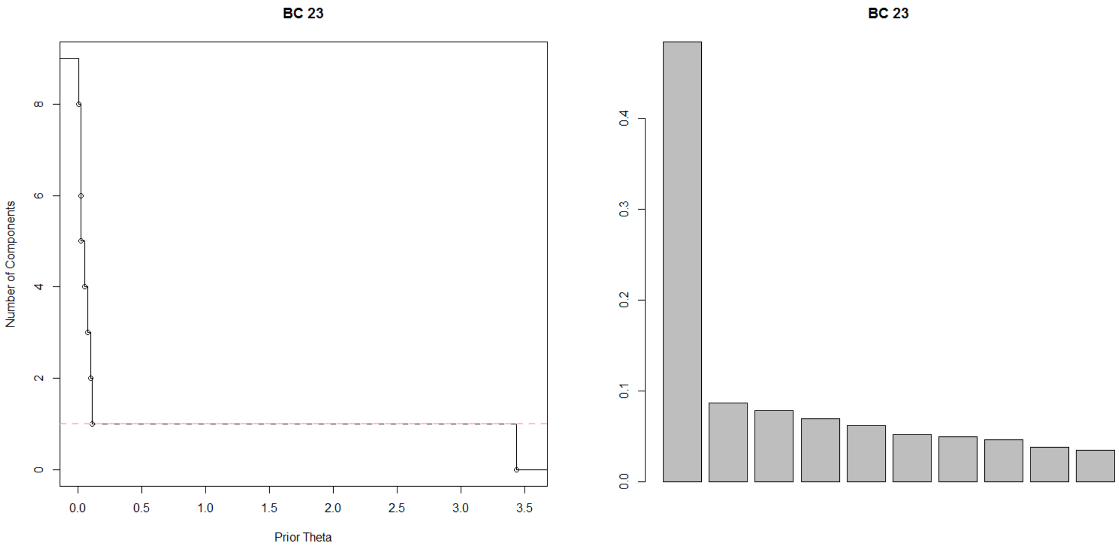

Supplementary Figure 38: The genes in biological component 23 form a one-dimensional space. (A) Auer-Gervini plot. (B) Scree plot.

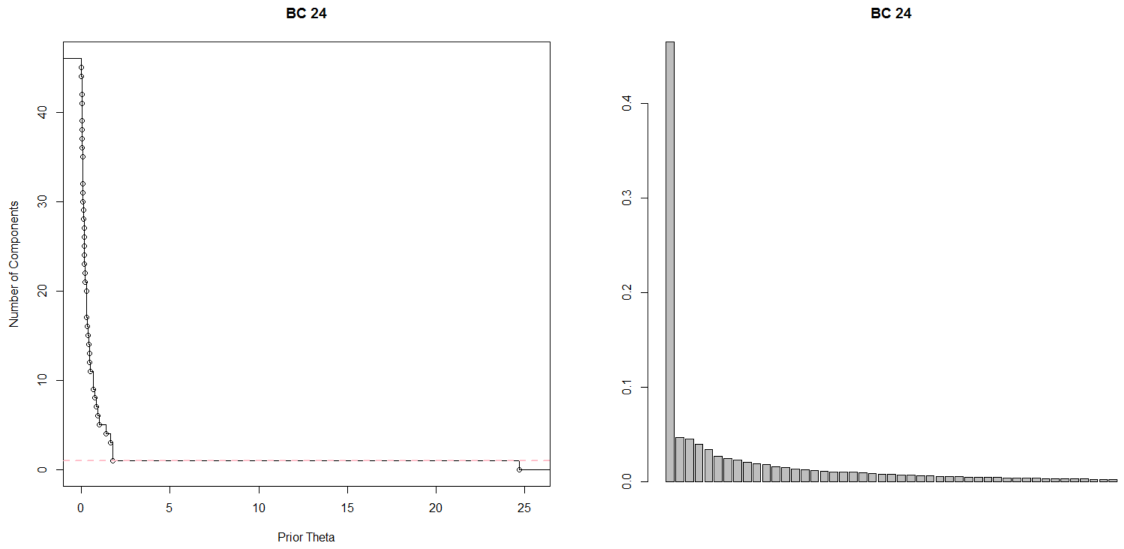

Supplementary Figure 39: The genes in biological component 24 form a one-dimensional space. (A) Auer-Gervini plot. (B) Scree plot.

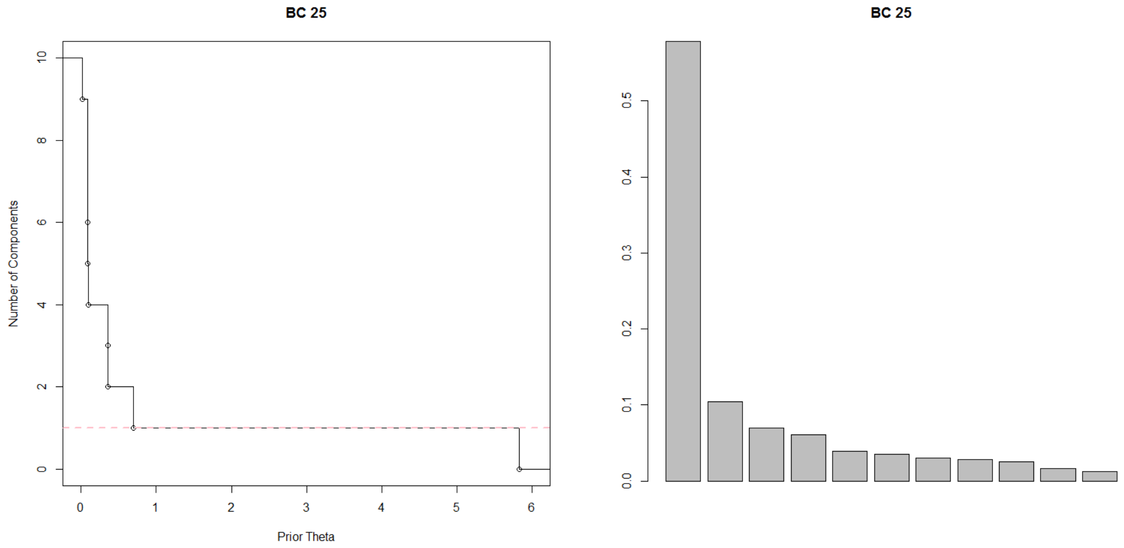

Supplementary Figure 40: The genes in biological component 25 form a one-dimensional space. (A) Auer-Gervini plot. (B) Scree plot.

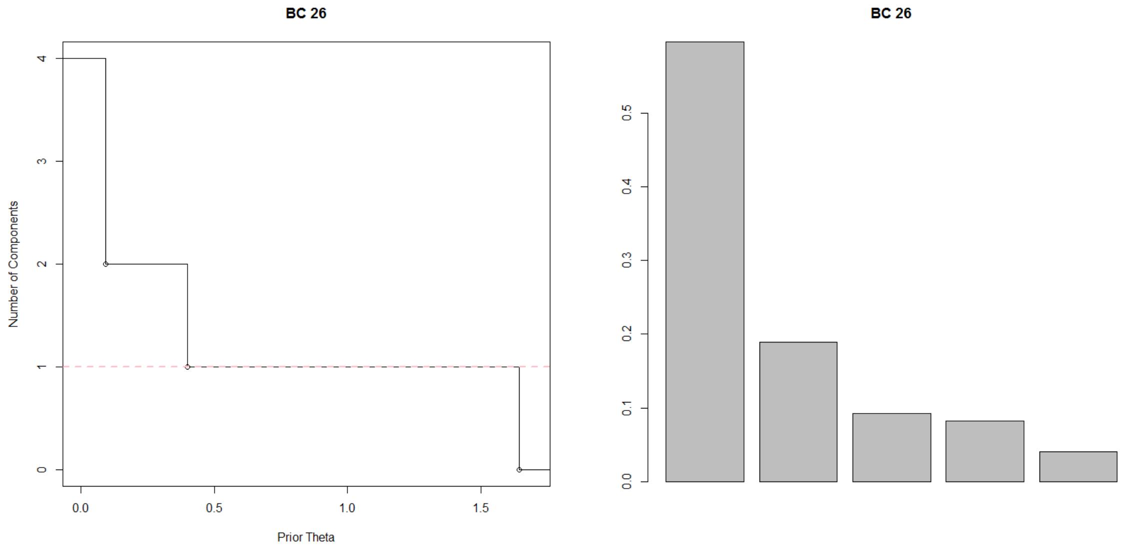

Supplementary Figure 41: The genes in biological component 26 form a one-dimensional space. (A) Auer-Gervini plot. (B) Scree plot.

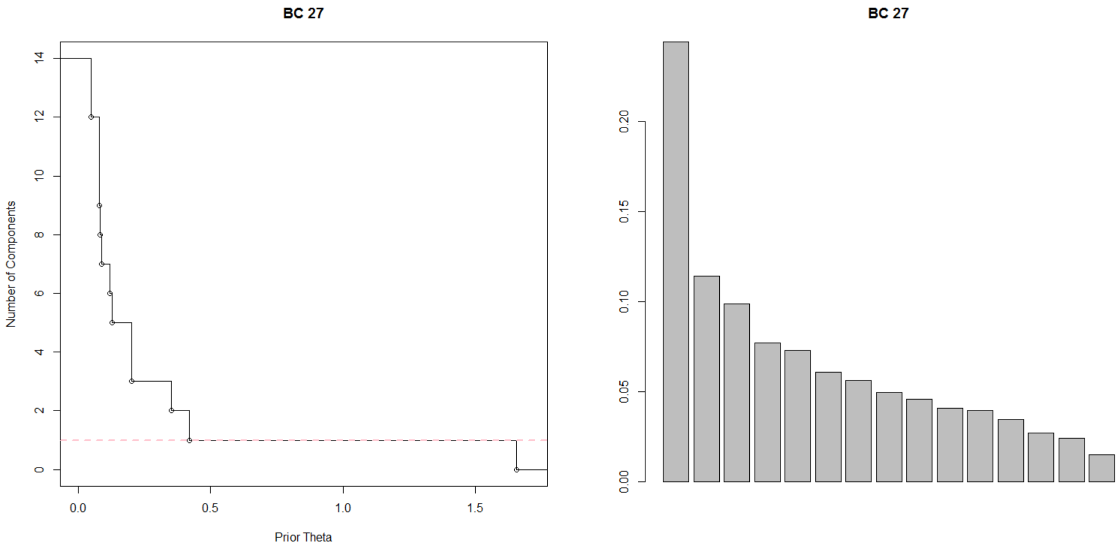

Supplementary Figure 42: The genes in biological component 27 form a one-dimensional space. (A) Auer-Gervini plot. (B) Scree plot.

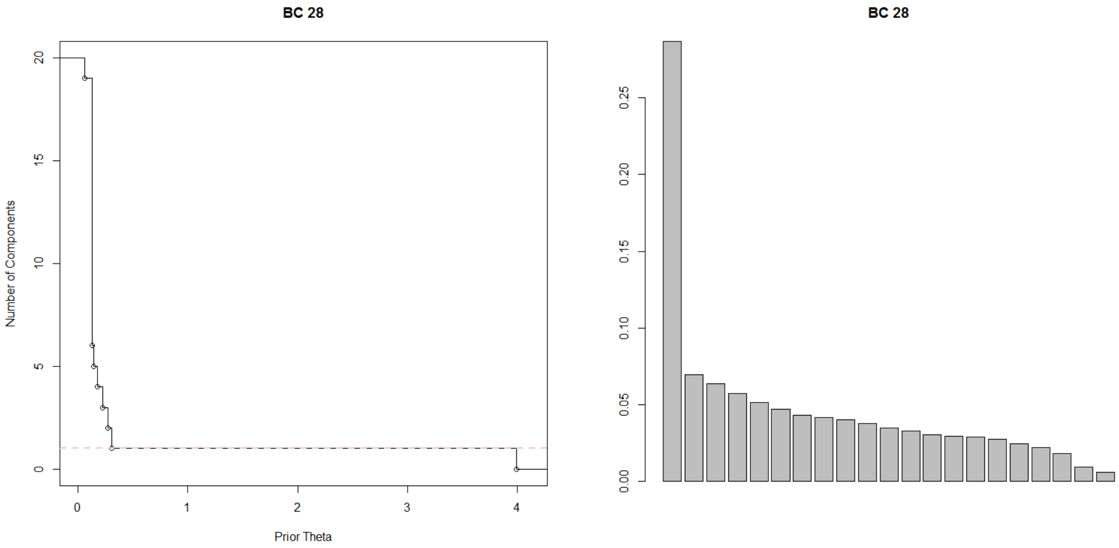

Supplementary Figure 43: The genes in biological component 28 form a one-dimensional space. (A) Auer-Gervini plot. (B) Scree plot.

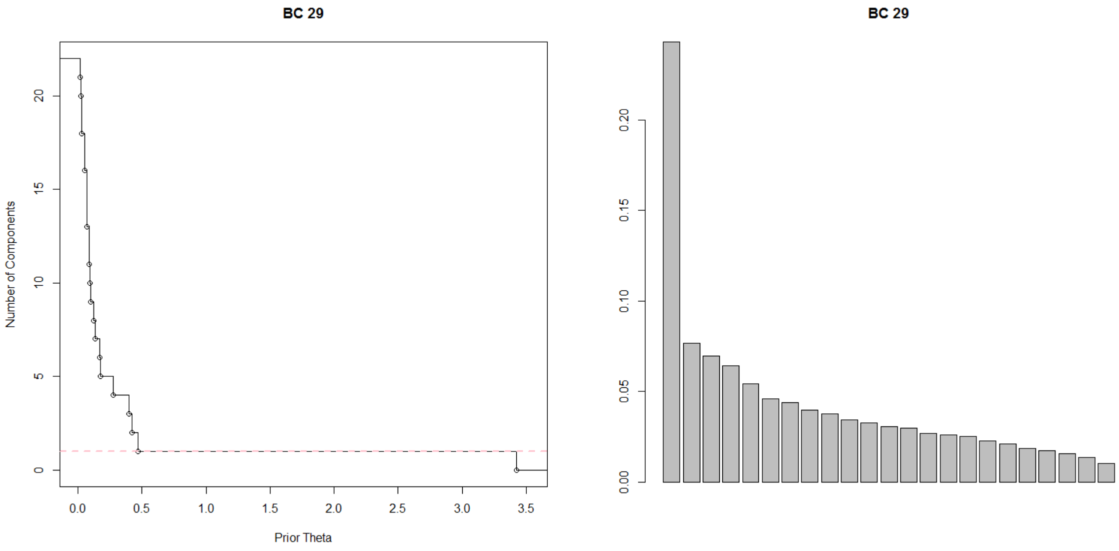

Supplementary Figure 44: The genes in biological component 29 form a one-dimensional space. (A) Auer-Gervini plot. (B) Scree plot.

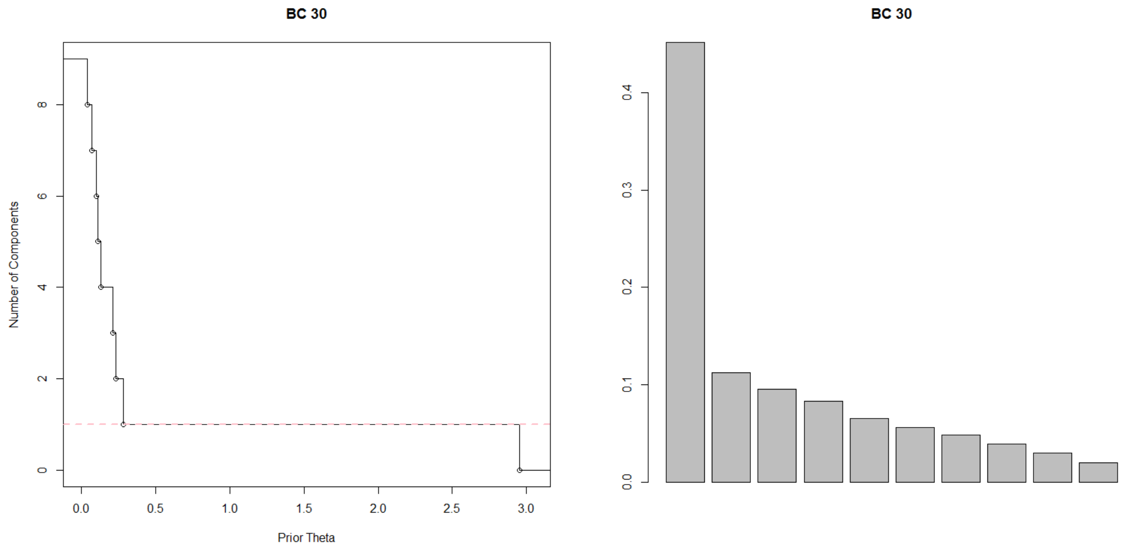

Supplementary Figure 45: The genes in biological component 30 form a one-dimensional space. (A) Auer-Gervini plot. (B) Scree plot.

4 Bean Plots Showing Cancer Type by Biological Component

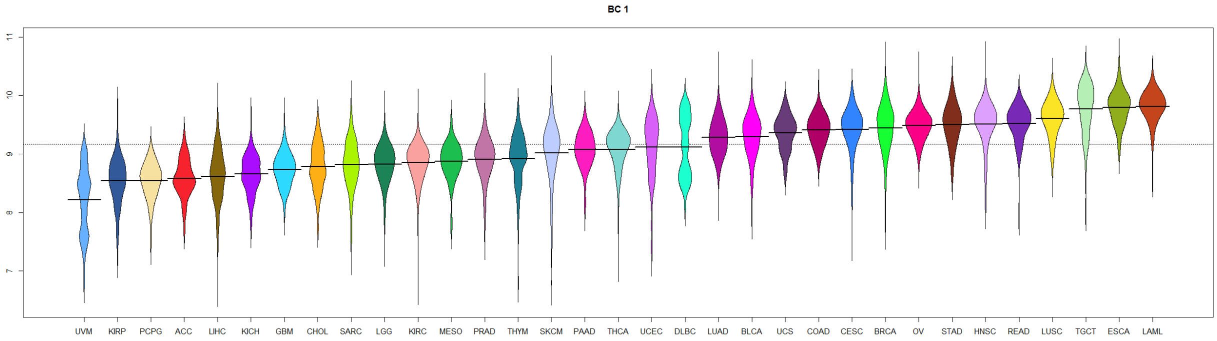

Supplementary Figure 46: Bean plot of cancer types for biological component 1.

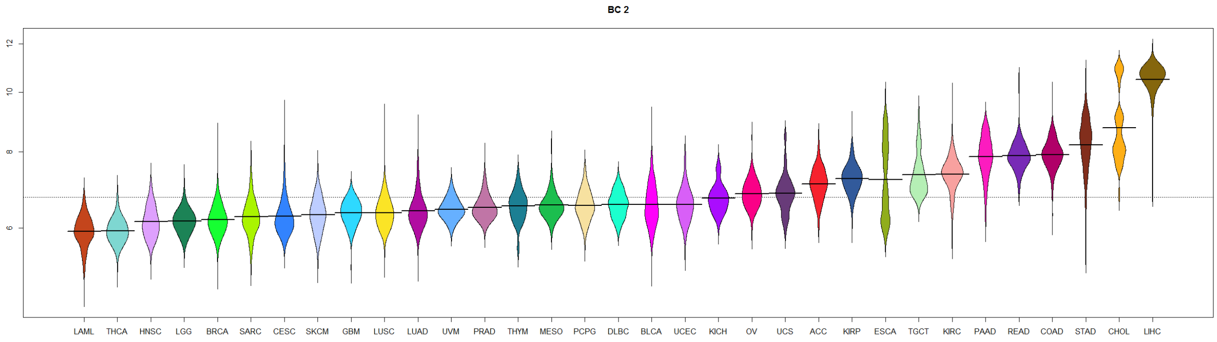

Supplementary Figure 47: Bean plot of cancer types for biological component 2.

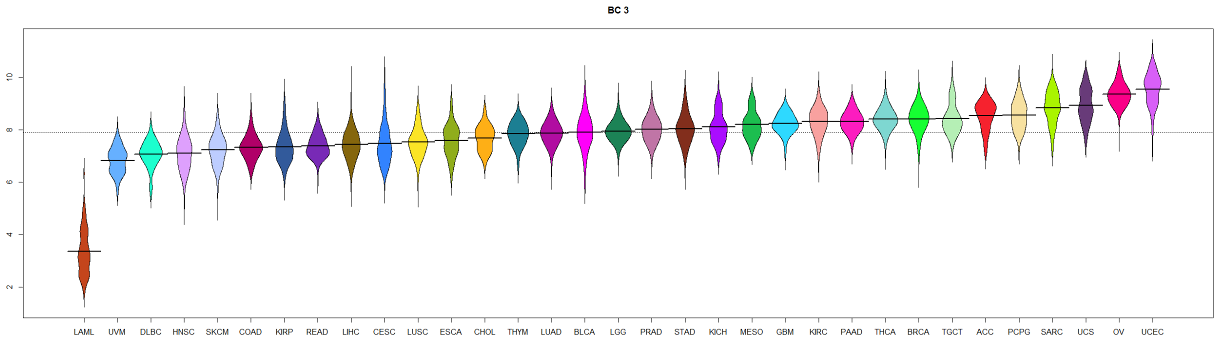

Supplementary Figure 48: Bean plot of cancer types for biological component 3.

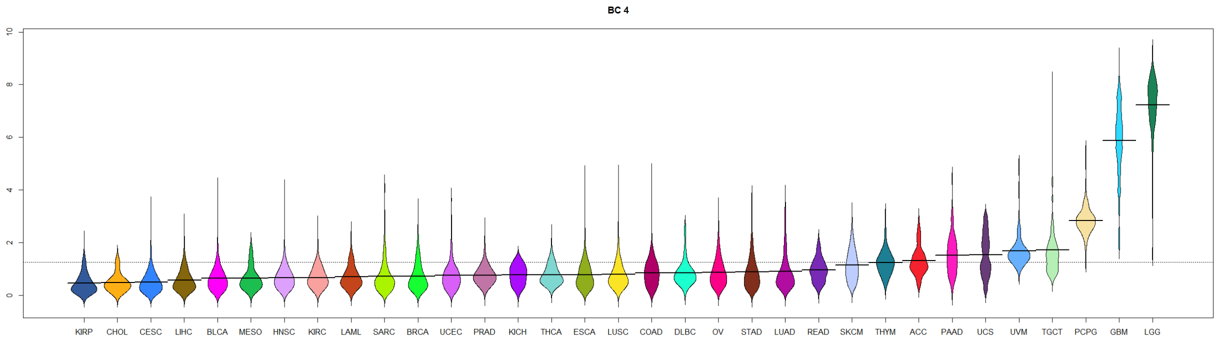

Supplementary Figure 49: Bean plot of cancer types for biological component 4.

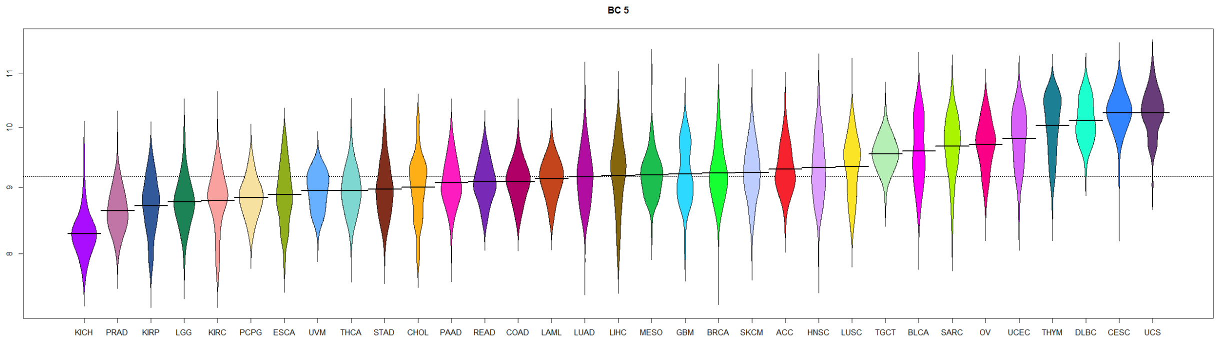

Supplementary Figure 50: Bean plot of cancer types for biological component 5.

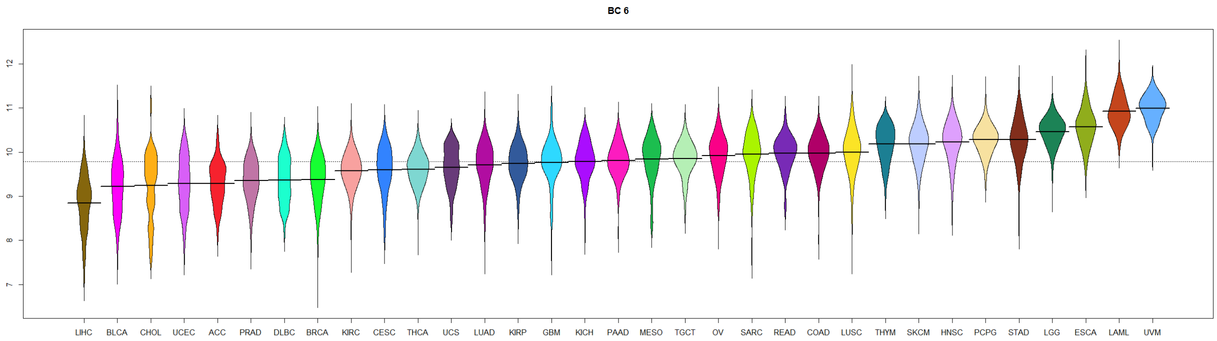

Supplementary Figure 51: Bean plot of cancer types for biological component 6.

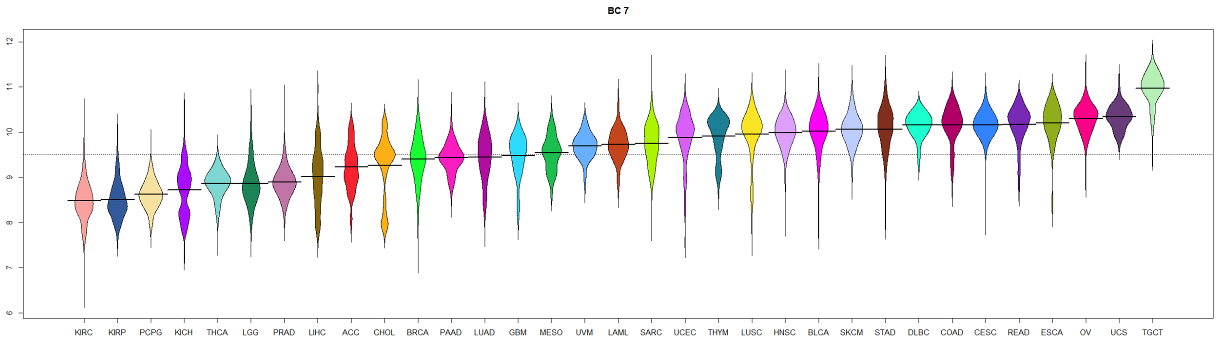

Supplementary Figure 52: Bean plot of cancer types for biological component 7.

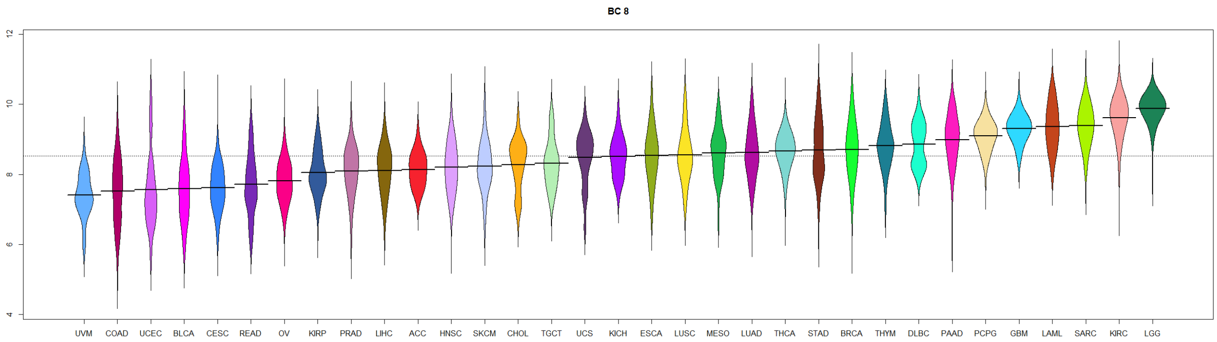

Supplementary Figure 53: Bean plot of cancer types for biological component 8.

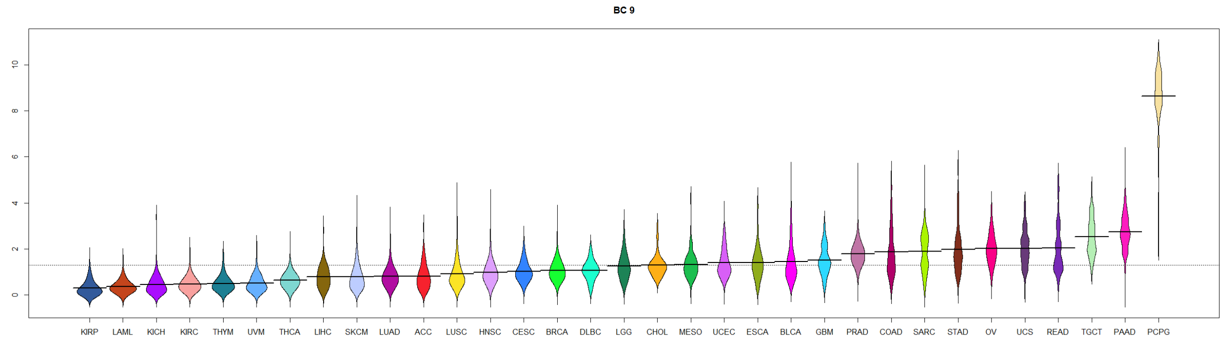

Supplementary Figure 54: Bean plot of cancer types for biological component 9.

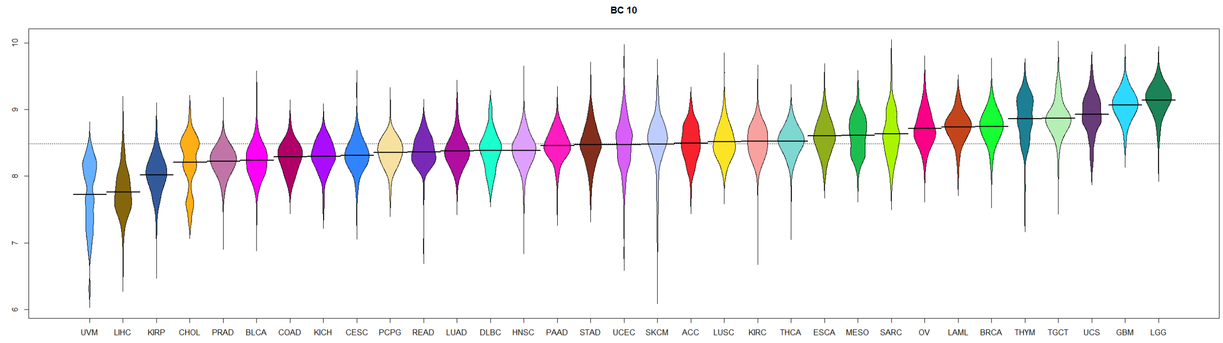

Supplementary Figure 55: Bean plot of cancer types for biological component 10.

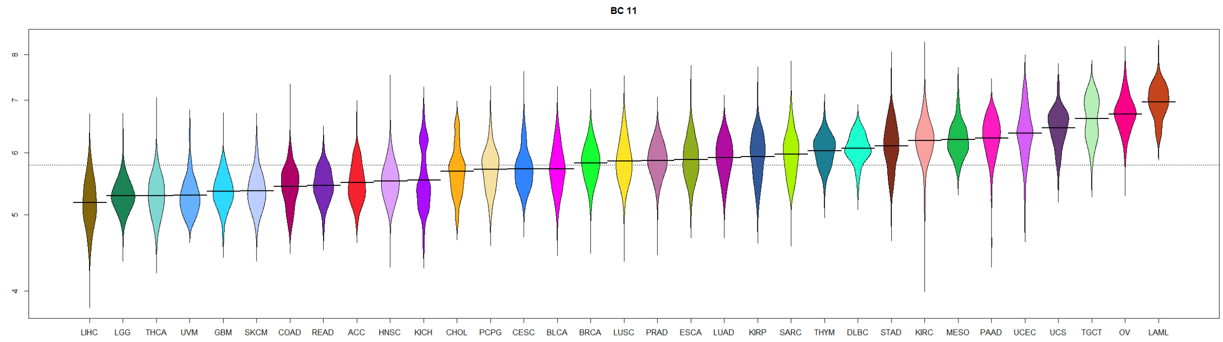

Supplementary Figure 56: Bean plot of cancer types for biological component 11.

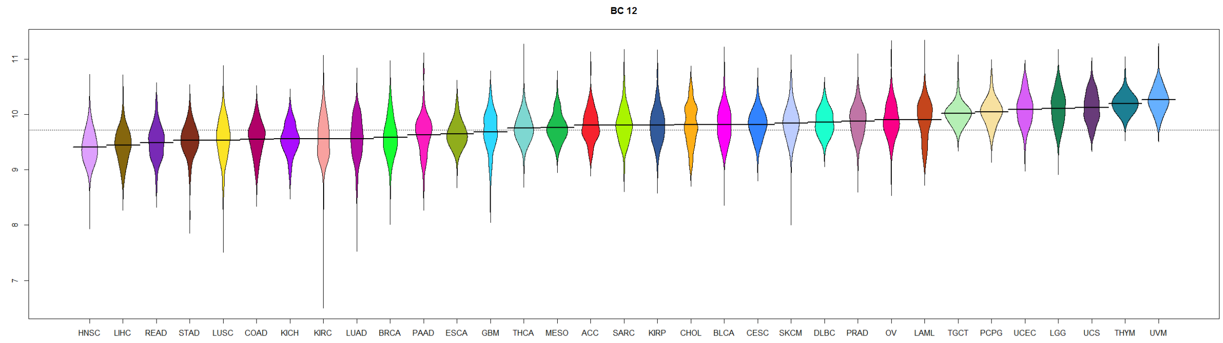

Supplementary Figure 57: Bean plot of cancer types for biological component 12.

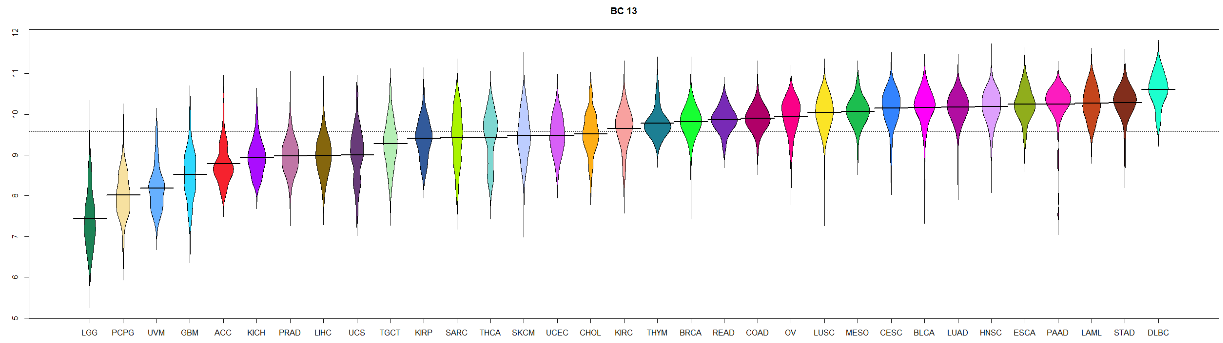

Supplementary Figure 58: Bean plot of cancer types for biological component 13.

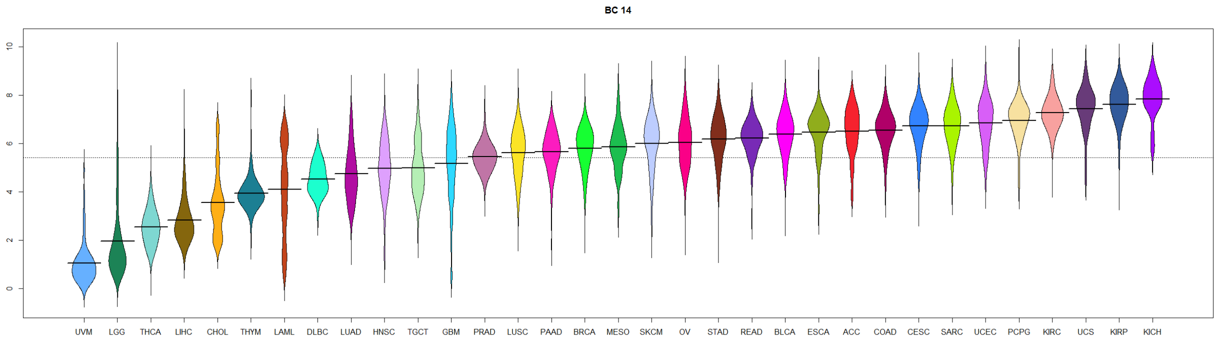

Supplementary Figure 59: Bean plot of cancer types for biological component 14.

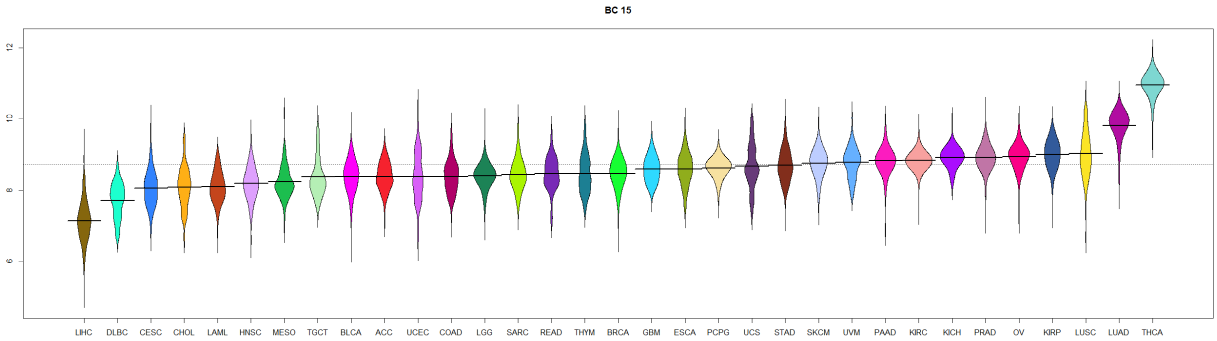

Supplementary Figure 60: Bean plot of cancer types for biological component 15.

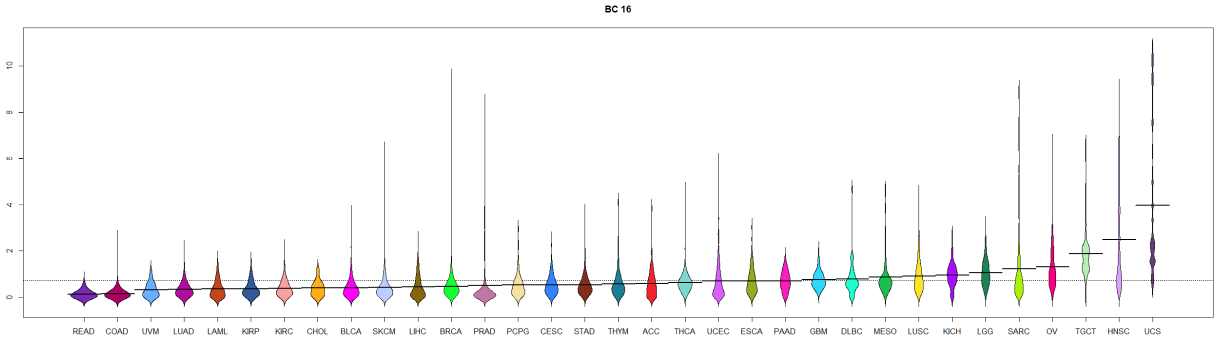

Supplementary Figure 61: Bean plot of cancer types for biological component 16.

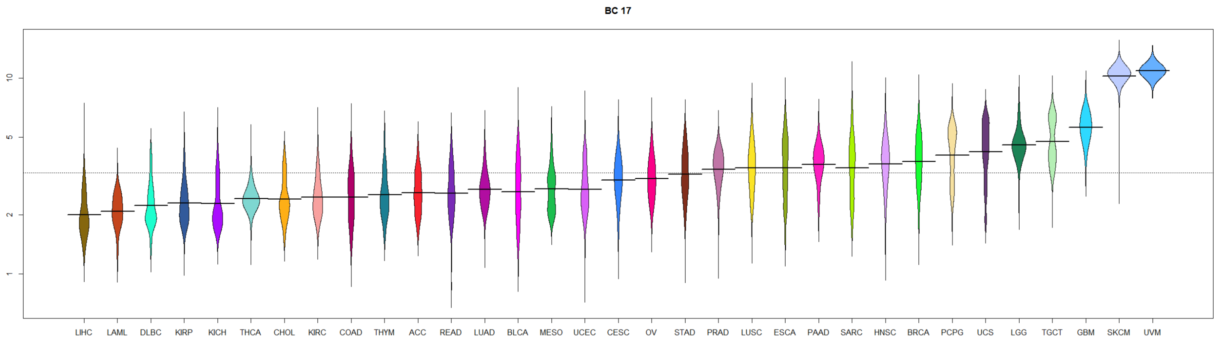

Supplementary Figure 62: Bean plot of cancer types for biological component 17.

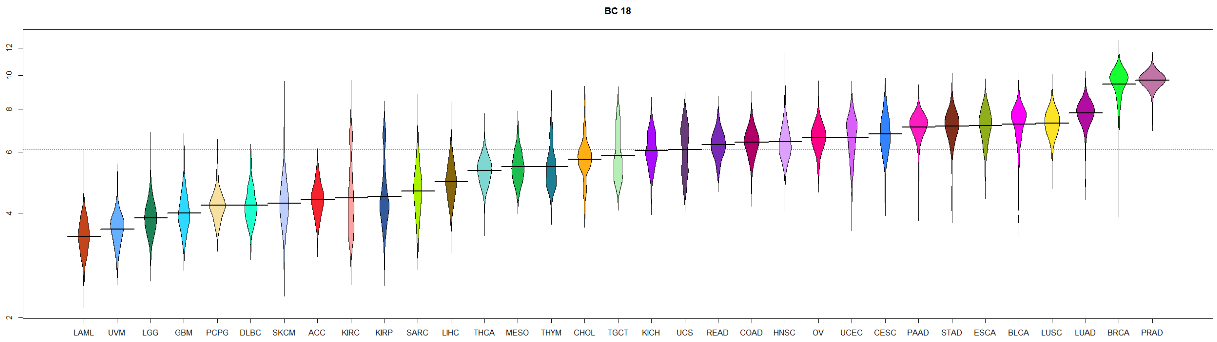

Supplementary Figure 63: Bean plot of cancer types for biological component 18.

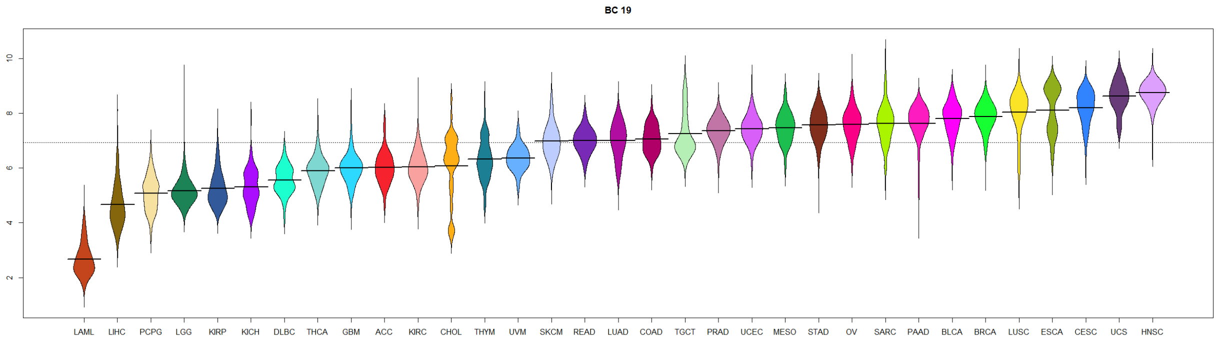

Supplementary Figure 64: Bean plot of cancer types for biological component 19.

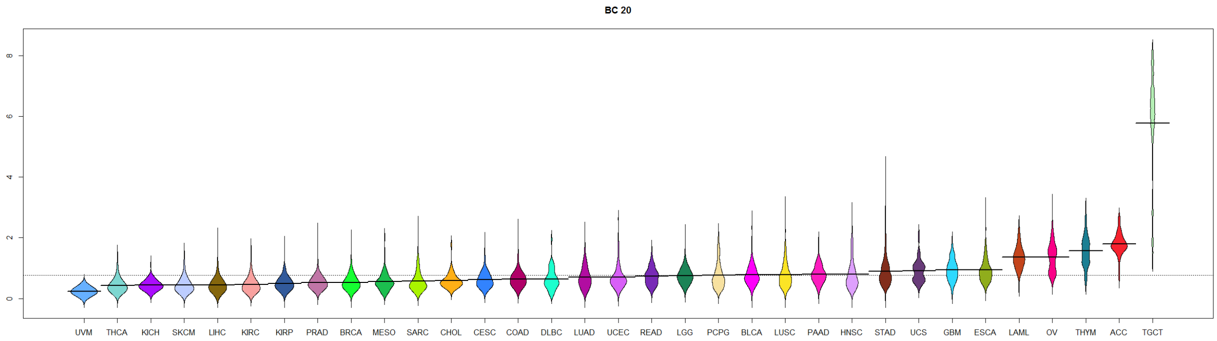

Supplementary Figure 65: Bean plot of cancer types for biological component 20.

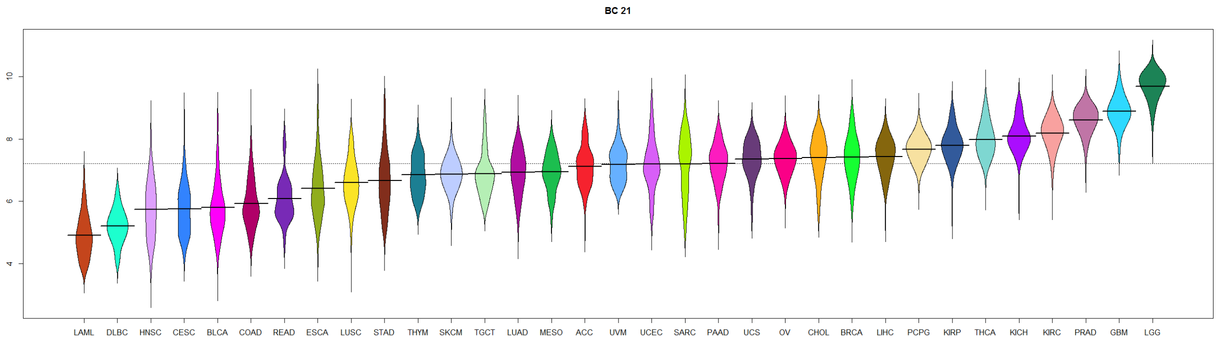

Supplementary Figure 66: Bean plot of cancer types for biological component 21.

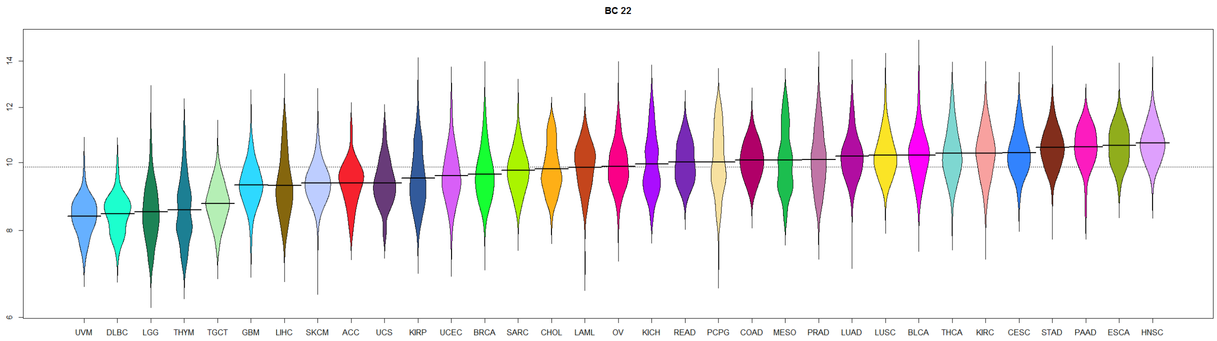

Supplementary Figure 67: Bean plot of cancer types for biological component 22.

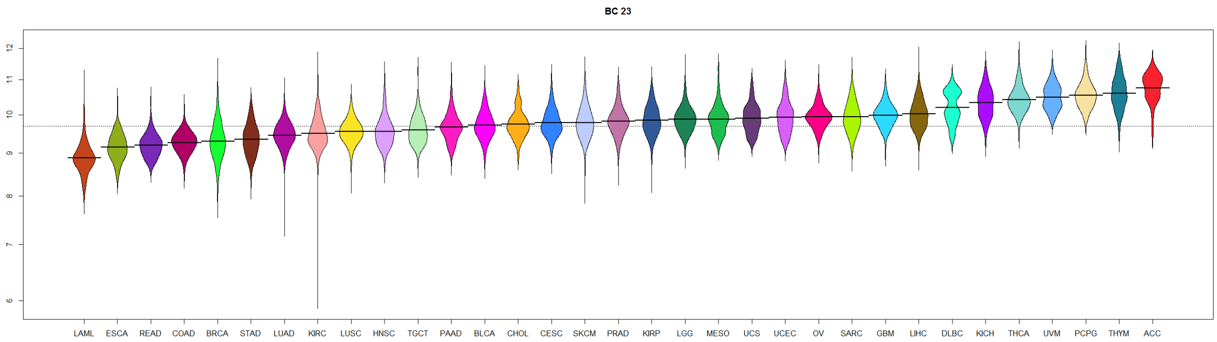

Supplementary Figure 68: Bean plot of cancer types for biological component 23.

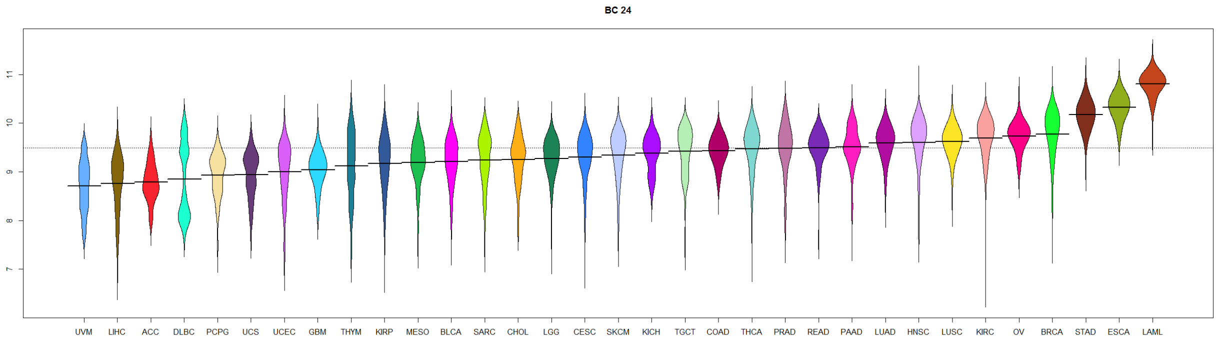

Supplementary Figure 69: Bean plot of cancer types for biological component 24.

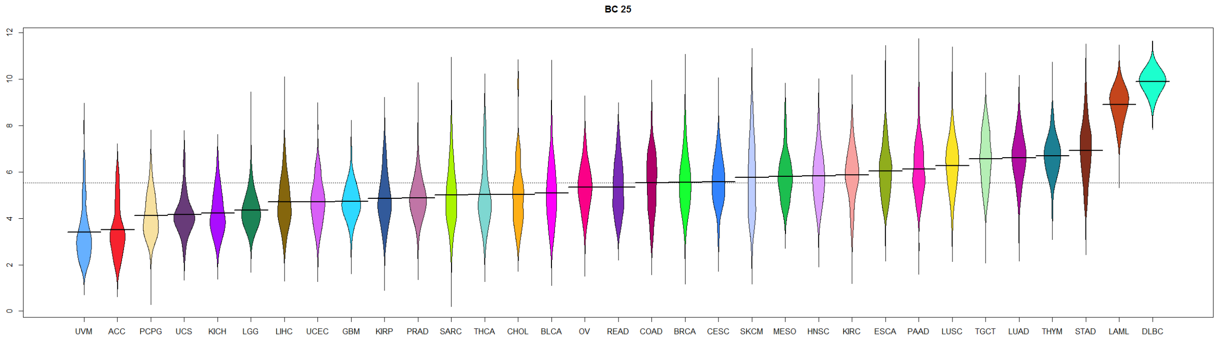

Supplementary Figure 70: Bean plot of cancer types for biological component 25.

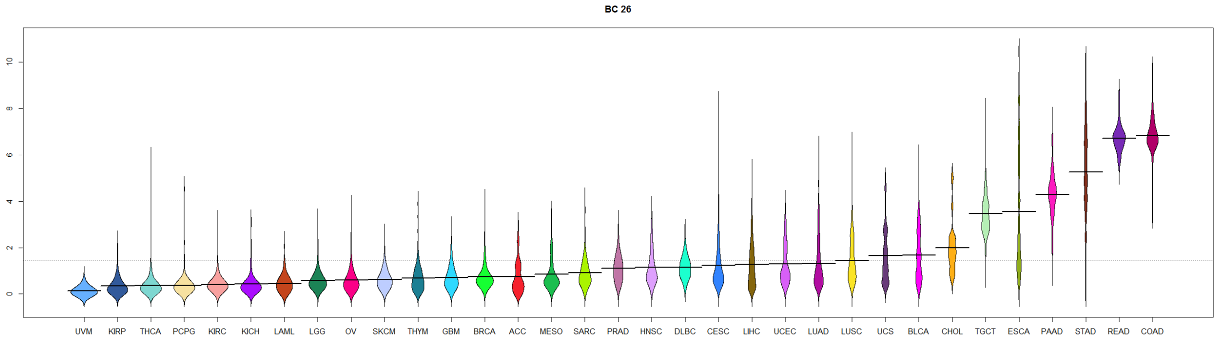

Supplementary Figure 71: Bean plot of cancer types for biological component 26.

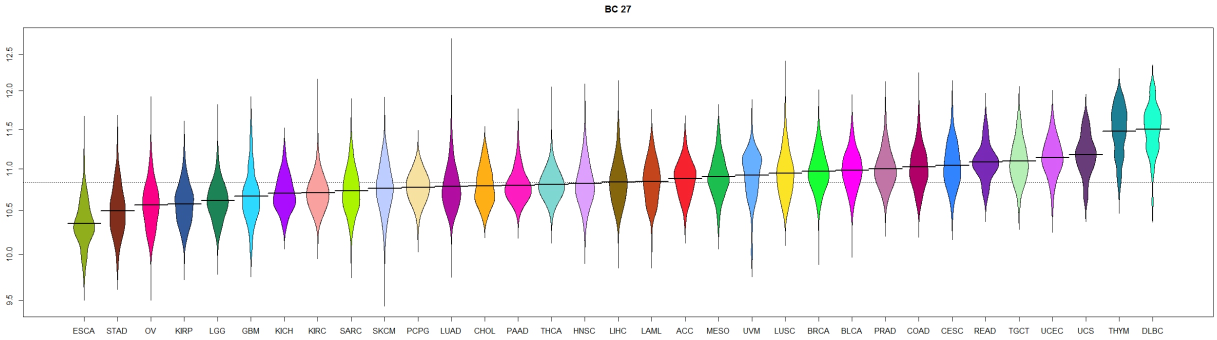

Supplementary Figure 72: Bean plot of cancer types for biological component 27.

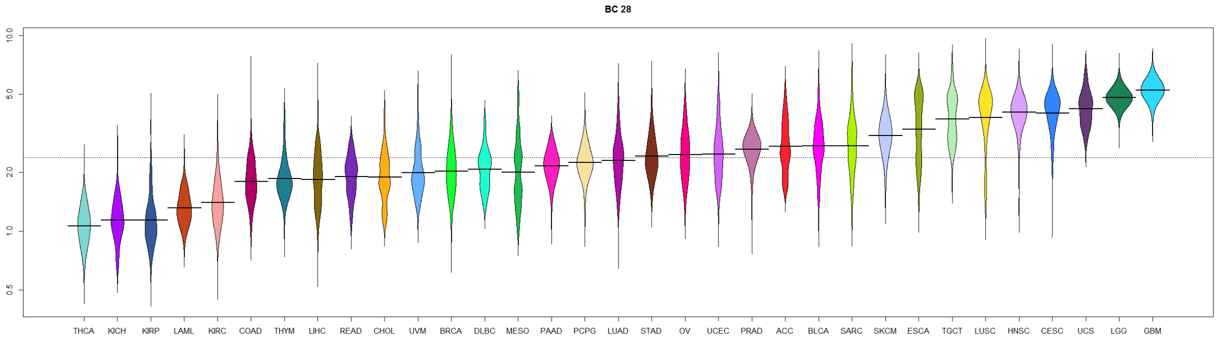

Supplementary Figure 73: Bean plot of cancer types for biological component 28.

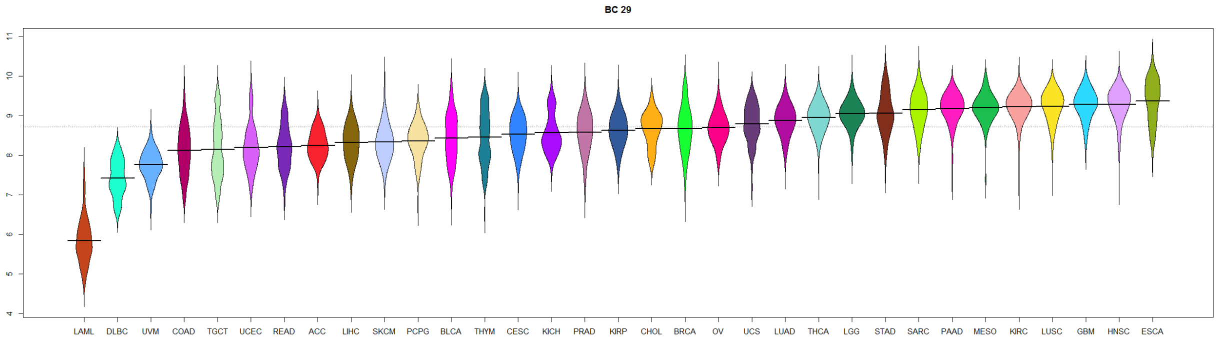

Supplementary Figure 74: Bean plot of cancer types for biological component 29.

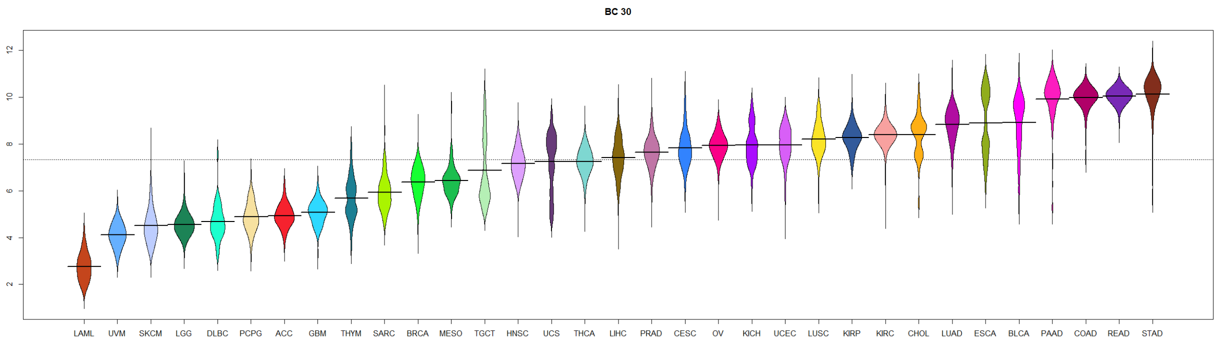

Supplementary Figure 75: Bean plot of cancer types for biological component 30.

5 Supplementary Tables

Note: The numbering of supplementary tables starts with zero instead of one in order to allow tables 1–30 to match biological components 1–30.

Supplementary Table 0: Transcription factor clusters. The Excel spreadsheet `SuppTable0.xlsx` contains a list of the 486 transcription factors used in this analysis, along with a column indicating which cluster or *biological component* each transcription factor is assigned to.

Supplementary Table 1: Results of ToppGene analysis of biological component BC 1. For each category of gene sets studied at the ToppGene web site, we retain at most 10 significant elements that are related to the set of genes correlated with BC 1.

Note: The descriptions of Supplementary Tables 2–29 are identical to that of Supplementary Table 1. The only change is that each table is associated with the biological component of the same number. All thirty Supplementary Tables can be found as separate worksheets inside the Excel spreadsheet `SuppTables.xlsx`
